# The Janus transcription factor HapX controls fungal adaptation to both iron starvation and iron excess

**DOI:** 10.15252/embj.201489468

**Published:** 2014-08-04

**Authors:** Fabio Gsaller, Peter Hortschansky, Sarah R Beattie, Veronika Klammer, Katja Tuppatsch, Beatrix E Lechner, Nicole Rietzschel, Ernst R Werner, Aaron A Vogan, Dawoon Chung, Ulrich Mühlenhoff, Masashi Kato, Robert A Cramer, Axel A Brakhage, Hubertus Haas

**Affiliations:** 1 Division of Molecular Biology, Biocenter, Innsbruck Medical UniversityInnsbruck, Austria; 2 Department of Molecular and Applied Microbiology, Leibniz Institute for Natural Product Research and Infection Biology (HKI)Jena, Germany; 3 Department of Microbiology and Immunology, Geisel School of Medicine at DartmouthHanover, NH, USA; 4 Friedrich Schiller UniversityJena, Germany; 5 Institut für Zytobiologie und Zytopathologie, Philipps-Universität MarburgMarburg, Germany; 6 Division of Biological Chemistry, Biocenter, Innsbruck Medical UniversityInnsbruck, Austria; 7 Department of Biology, McMaster UniversityHamilton, ON, Canada; 8 Faculty of Agriculture, Meijo UniversityNagoya, Japan

**Keywords:** fungi, iron regulation, sensing, siderophores, transcription factor complex

## Abstract

Balance of physiological levels of iron is essential for every organism. In *Aspergillus fumigatus* and other fungal pathogens*,* the transcription factor HapX mediates adaptation to iron limitation and consequently virulence by repressing iron consumption and activating iron uptake. Here, we demonstrate that HapX is also essential for iron resistance via activating vacuolar iron storage. We identified HapX protein domains that are essential for HapX functions during either iron starvation or high-iron conditions. The evolutionary conservation of these domains indicates their wide-spread role in iron sensing. We further demonstrate that a HapX homodimer and the CCAAT-binding complex (CBC) cooperatively bind an evolutionary conserved DNA motif in a target promoter. The latter reveals the mode of discrimination between general CBC and specific HapX/CBC target genes. Collectively, our study uncovers a novel regulatory mechanism mediating both iron resistance and adaptation to iron starvation by the same transcription factor complex with activating and repressing functions depending on ambient iron availability.

## Introduction

The redox-active metal iron is an indispensable cofactor in a variety of essential cellular processes such as oxidative phosphorylation, biosynthesis of numerous metabolites, and detoxification of oxidative stress. Paradoxically, the same redox property makes this metal potentially toxic by causing oxidative stress (Halliwell & Gutteridge, 1984; Lin *et al*, 2011). Thus, iron homeostasis requires precise regulation of iron uptake and storage to satisfy the cellular needs but to avoid toxic iron excess.

In *Aspergillus fumigatus,* iron homeostasis is maintained by two central transcription factors, which are interconnected in a negative transcriptional feed-back loop: the GATA-factor SreA and the bZIP-factor HapX (Haas, 2012). During iron sufficiency, SreA represses iron uptake, including reductive iron assimilation and siderophore-mediated iron uptake, to avoid toxic effects (Schrettl *et al*, 2008). During iron starvation, HapX activates siderophore-mediated iron acquisition and represses iron-consuming pathways, including heme biosynthesis and respiration, to spare iron (Schrettl *et al*, 2010). As shown in *Aspergillus nidulans*, HapX functions via physical interaction with the CCAAT-binding complex (CBC) (Hortschansky *et al*, 2007). The CBC is a heterotrimeric DNA-binding complex, which is conserved in all eukaryotes. In *A. nidulans*, inactivation of either one of its subunits, phenocopied HapX inactivation with respect to defects in adaptation to iron starvation (Hortschansky *et al*, 2007). However, the CBC has HapX-independent functions in *Aspergillus* spp. (Kato, 2005; Thon *et al*, 2010). Humans lack HapX and genome-wide identification resulted in 5,000–15,000 CBC-binding sites depending on the type of human cell analyzed (Fleming *et al*, 2013).

Deficiency in HapX, but not SreA, attenuates virulence of *A. fumigatus* in murine models of aspergillosis (Schrettl *et al*, 2008, 2010), which emphasizes the crucial role of adaptation to iron limitation in pathogenicity. With the exception of *Saccharomyces cerevisiae* and closely related *Saccharomycotina* species, most fungal species possess orthologs to SreA and HapX (Haas *et al*, 2008; Kaplan & Kaplan, 2009). The important role of HapX orthologs in virulence is conserved in *Candida albicans*, *Cryptococcus neoformans,* and *Fusarium oxysporum* (Jung *et al*, 2010; Chen *et al*, 2011; Hsu *et al*, 2011; Lopez-Berges *et al*, 2012). Both SreA and HapX appear to be regulated post-translationally by iron, blocking HapX function and activating SreA function (Haas *et al*, 1999; Hortschansky *et al*, 2007). In *Schizosaccharomyces pombe*, post-translational iron sensing by the HapX and SreA orthologs involves the monothiol glutaredoxin Grx4 (Labbe *et al*, 2013).

Recently, iron resistance of *A. fumigatus* was shown to involve SreA-mediated repression of iron uptake and vacuolar iron storage mediated by the vacuolar iron importer CccA (Gsaller *et al*, 2012). In *A. nidulans* and *A. fumigatus*, inactivation of both HapX and SreA is synthetically lethal underlining the critical role of iron homeostasis in cellular survival (Hortschansky *et al*, 2007; Schrettl *et al*, 2010). In agreement with their expression pattern and characterized mode of action, the detrimental effects of SreA or HapX inactivation identified so far were confined to growth during iron sufficiency or starvation, respectively, which does not explain the synthetic lethality of their inactivation. Here, we provide an explanation for this synthetic lethality by demonstrating that HapX mediates both repression of vacuolar iron storage during iron starvation and activation of vacuolar iron storage during iron excess, i.e. HapX displays inverse activities depending on the ambient iron availability. In line, we identified protein domains that are essential for mediating adaptation to iron starvation or iron excess, exclusively. Moreover, we demonstrate for the first time that HapX not only acts via protein–protein interaction with the CBC but also directly recognizes an evolutionary conserved motif in the *cccA* promoter. As the CBC has HapX/iron-independent targets, the latter data reveal the mechanism for discrimination of general CBC and specific HapX/CBC target genes.

## Results and Discussion

### HapX mediates iron resistance by activating CccA-mediated vacuolar iron storage

HapX functions were analyzed in *A. fumigatus* ATCC 46645 (Schrettl *et al*, 2010) and, to facilitate the studies, in its Δ*akuA*-derivative AfS77, which lacks non-homologous recombination (Hartmann *et al*, 2010). We did not observe any phenotypic differences between respective ATCC46645- and AfS77*-*derivative strains (data not shown). For clarity, however, the genetic background used is given for all experiments.

Previously, genome-wide transcriptional profiling revealed that during iron starvation HapX activates genes involved in iron acquisition (including siderophore transporter-encoding *mirB*) and represses the vacuolar iron transporter-encoding *cccA* as well as numerous genes involved in iron-consuming processes (see below) (Schrettl *et al*, 2010). CccA-mediated vacuolar iron storage was recently shown to mediate iron resistance (Gsaller *et al*, 2012). Consistently, the *cccA* transcript level is upregulated by iron and particularly by SreA-deficiency (Gsaller *et al*, 2012). The latter is consistent with SreA-deficiency increasing the cellular iron content (Schrettl *et al*, 2008) but also shows that transcriptional activation of *cccA* is mediated by an SreA-independent regulatory mechanism.

**Figure 1:**
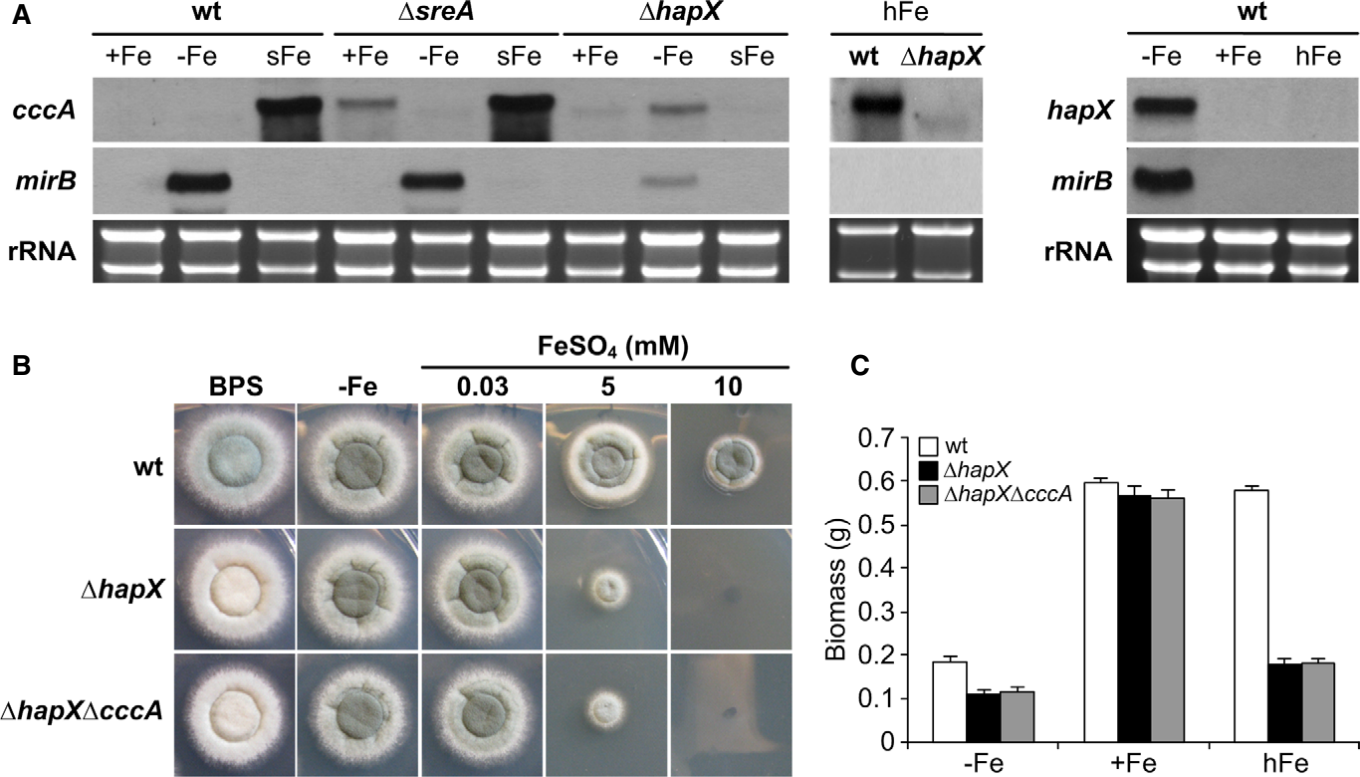
HapX is important for adaptation to both iron limitation and iron excess HapX represses *cccA* during iron starvation and activates *cccA* during iron excess. Northern analysis was performed with liquid cultures under conditions of iron starvation (−Fe), iron sufficiency (+Fe, 0.03 mM FeSO_4_), and high-iron availability (hFe, 3 mM FeSO_4_) at 37°C for 24 h or from mycelia shifted for 1 h from −Fe to +Fe (sFe).On agar plates, HapX-deficiency impairs sporulation on BPS-plates, and growth during iron excess. Growth pattern of wild-type (wt), Δ*hapX* and Δ*hapX*Δ*cccA* on solid minimal media containing the indicated iron concentration is shown after 48 h at 37°C. The greenish color of the fungal colonies originates from the spore pigment, and its decrease indicates reduced sporulation. The original size of fungal colony photographs is 2.3 × 2.3 cm in all figures.HapX-deficiency impairs submerged growth during both iron starvation and iron excess. Liquid biomass production was monitored after 24 h of growth at 37°C under the indicated iron availability. The data represent the mean ± standard deviation (SD) of biological triplicates. The difference between mutant and wild-type strains was statistically significant during −Fe and hFe but not +Fe (two-tailed, unpaired *t*-test; *P* < 0.05). Data information: The iron-sensitive phenotype of Δ*cccA* was previously analyzed in Gsaller *et al* (2012) and was further characterized in Fig2. Moreover, the response of *hapX* transcript levels to a 1-h shift from iron starvation to sufficiency (sFe) was analyzed in Fig4. Strains are derivatives of *A. fumigatus* AfS77.

Northern analysis demonstrated that HapX-deficiency (strain Δ*hapX)* impairs not only repression of *cccA* during iron starvation but also induction of *cccA* during a 1-h shift from iron starvation to iron sufficiency as well as during growth in high-iron medium (Fig1A). As shown previously (Schrettl *et al*, 2010), HapX-deficiency caused downregulation of *mirB* during iron starvation, but did not affect repression of *mirB* by iron (Fig1A).

The role of HapX in transcriptional control of *cccA* during iron excess implicated a role of HapX in iron detoxification. In agreement, HapX-deficiency not only decreased sporulation on agar plates in the presence of the iron starvation-inducing, iron-specific chelator bathophenanthroline disulfonate (BPS) and decreased biomass production in liquid cultures during iron starvation, as shown previously (Schrettl *et al*, 2010), but also dramatically decreased growth on solid and in liquid high-iron media (Fig1B and C). As reported previously (Schrettl *et al*, 2010), HapX-deficiency did affect neither growth rate nor sporulation under iron-replete conditions.

A mutant strain lacking both HapX and CccA, Δ*hapX*Δ*cccA*, displayed the same growth pattern as Δ*hapX* on solid and in liquid media (Fig1B and C). The epistasis of HapX- to CccA-deficiency strongly suggests that lack of *cccA* expression is responsible for the Δ*hapX* growth defect during iron excess.

Taken together, HapX acts as a Janus-type transcription factor mediating both repression and activation of *cccA* and consequently vacuolar iron storage depending on the ambient iron availability.

### HapX additionally controls CccA-independent mechanisms involved in iron detoxification

**Figure 2:**
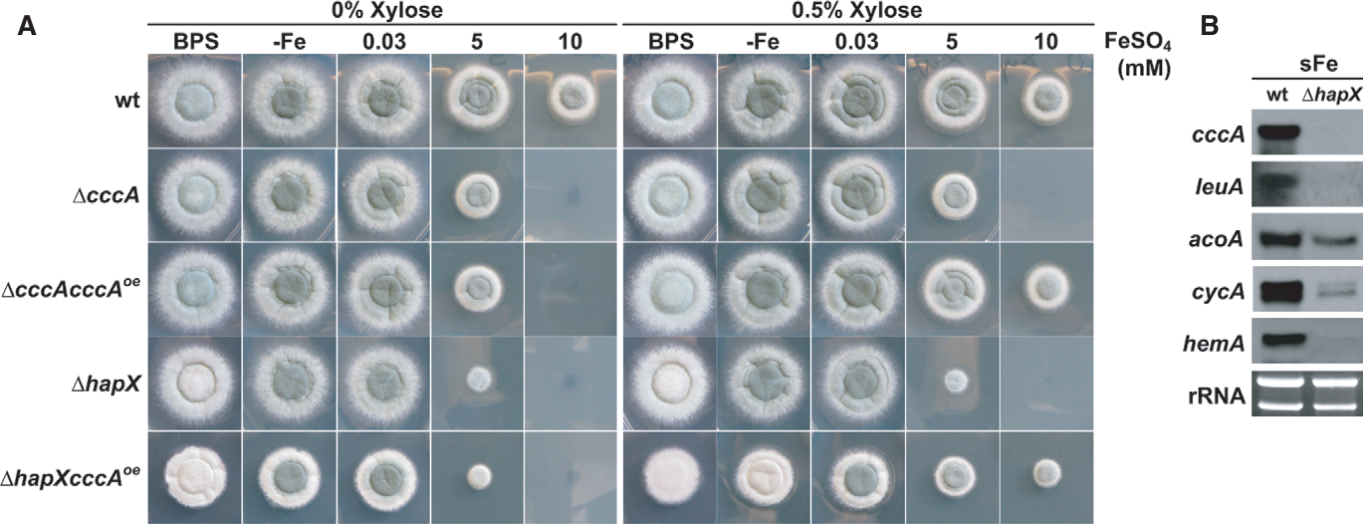
HapX-deficiency renders *A. fumigatus* more susceptible to iron toxicity than CccA-deficiency and impairs induction of genes involved in iron-consuming processes Strains were grown on solid minimal medium with the given iron availability under *xylP*-driven *cccA*^*OE*^ non-inducible (0% xylose) and inducible (0.5% xylose) conditions for 48 h at 37°C.Northern blot analysis was performed after liquid growth for 24 h at 37°C under iron limitation and a subsequent 1-h shift to iron sufficiency (sFe). rRNA is shown as a control for RNA quantity and quality. Data information: Strains are derivatives of *A. fumigatus *ATCC 46645.

Notably, HapX-deficiency rendered *A. fumigatus* more susceptible to iron toxicity than CccA-deficiency on solid (Fig2A) and in liquid (Supplementary Table S1) high-iron media. These data indicate that HapX is also required for the activity of iron detoxification mechanisms other than CccA-mediated iron storage. In support, conditional expression of *cccA* using the xylose-inducible *xylP* promoter (Zadra *et al*, 2000; Gsaller *et al*, 2012) increased iron resistance of Δ*hapX* under inducing but not repressing conditions (Fig2A; compare strains Δ*hapX* and Δ*hapXcccA*^*OE*^). However, the radial growth of this strain did not reach that of the wild-type or the Δ*cccAcccA*^*OE*^ strain (a *cccA* deletion mutant expressing *cccA* under control of the inducible *xylP* promoter). Compared to Δ*hapX*, Δ*hapXcccA*^*OE*^ also displayed a significant decrease in growth and sporulation on BPS- and low-iron agar plates, demonstrating that activation of vacuolar iron storage is particularly detrimental in a HapX-deficient background.

As previously indicated by genome-wide transcriptional profiling (Schrettl *et al*, 2010), apart from *cccA* numerous other genes involved in iron-consuming processes are repressed by HapX during iron starvation. Northern analysis revealed that in a 1-h shift from iron-limited to iron-replete conditions, which reflects short-term iron excess, HapX-deficiency impairs the transcriptional activation not only of *cccA* but also of genes encoding iron-consuming functions (Fig2B). These proteins include the iron–sulfur cluster-containing LeuA (α-isopropylmalate isomerase) and AcoA (aconitase), involved in leucine biosynthesis and the TCA cycle, respectively, the heme-containing CycA (cytochrome *c*) involved in respiration, and the heme biosynthesis protein HemA (δ-aminolevulinic acid synthase). These data indicate that HapX might help to detoxify iron excess via general upregulation of iron-dependent proteins and processes. In agreement with the iron-detoxifying activity of iron-dependent proteins, overexpression of iron–sulfur cluster enzymes has been shown to attenuate iron toxicity in *S. cerevisiae* (Li *et al*, 2011).

### HapX levels are significantly higher during iron starvation compared to sufficiency or excess of iron

**Figure 3:**
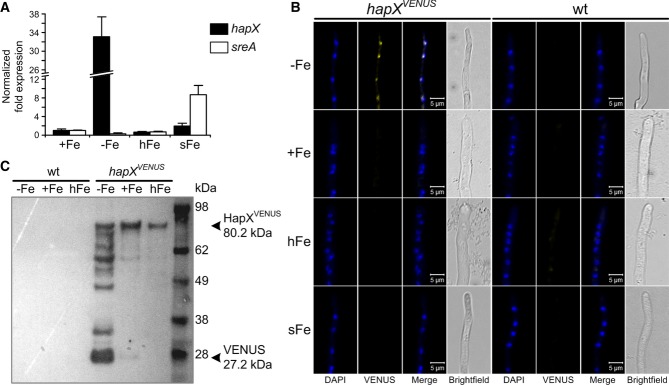
HapX production decreases during ambient and high-iron availability qRT-PCR revealing iron-dependent *sreA* and *hapX* transcript abundance. Transcript levels of *hapX* and *sreA* were determined during iron starvation (−Fe), iron sufficiency (+Fe, 0.03 mM), iron excess (hFe, 3 mM) and after a 1-h shift from iron starvation to iron sufficiency (sFe) and normalized to that of γ-actin (AFUA_6G04740) using the 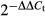 method. Data represent the mean ± SD of two biological and three PCR technical replicates and are presented relative to the transcript levels during iron sufficiency. All differences found are statistically significant with exception of the *hapX* transcript level during iron sufficiency compared to high-iron conditions (two-tailed, unpaired *t*-test; *P* < 0.05).In epifluorescence microscopy, HapX^VENUS^ is detectable in the nuclei only during iron starvation. 10^4^ spores of the respective strain were grown in 24-well plates in liquid media at 37°C for 18 h. DAPI was used for staining of nuclei.Western blot analysis after GFP-trap enrichment revealing significantly increased HapX^VENUS^ production during iron starvation. The molecular mass of HapX^VENUS^ is 80.2 kDa (27.2 kDa Venus + 53 kDa HapX). Data information: Strains are derivatives of *A. fumigatus* AfS77.

In contrast to iron sufficiency, *A. fumigatus hapX* and its orthologs in *A. nidulans, F. oxysporum, C. albicans, S. pombe,* and *C. neoformans*, are transcriptionally upregulated by iron starvation (Mercier *et al*, 2006; Hortschansky *et al*, 2007; Jung *et al*, 2010; Schrettl *et al*, 2010; Hsu *et al*, 2011; Lopez-Berges *et al*, 2012). Remarkably, Northern analysis did not detect *hapX* transcripts during iron excess despite the HapX requirement under this condition (Fig1A). To increase the sensitivity of transcript detection, *hapX* transcript levels were quantified by qRT-PCR and compared to that of *sreA* (Fig3A). This analysis confirmed highest *hapX* expression during iron starvation, i.e. 33-fold higher compared to iron sufficiency, and 17-fold downregulation after a 1-h shift from iron starvation to iron sufficiency. As reported previously (Schrettl *et al*, 2008), *sreA* expression was increased (about threefold) during iron sufficiency compared to iron starvation and highly upregulated (about 29-fold) during a 1-h shift from iron starvation to iron sufficiency. During iron excess, a condition in which SreA was previously found to be important for iron resistance (Schrettl *et al*, 2008; Gsaller *et al*, 2012), the *sreA* transcript level was about threefold increased compared to that of *hapX* (data not shown), i.e. *hapX* was clearly expressed, although below the Northern sensitivity level.

**Figure 4:**
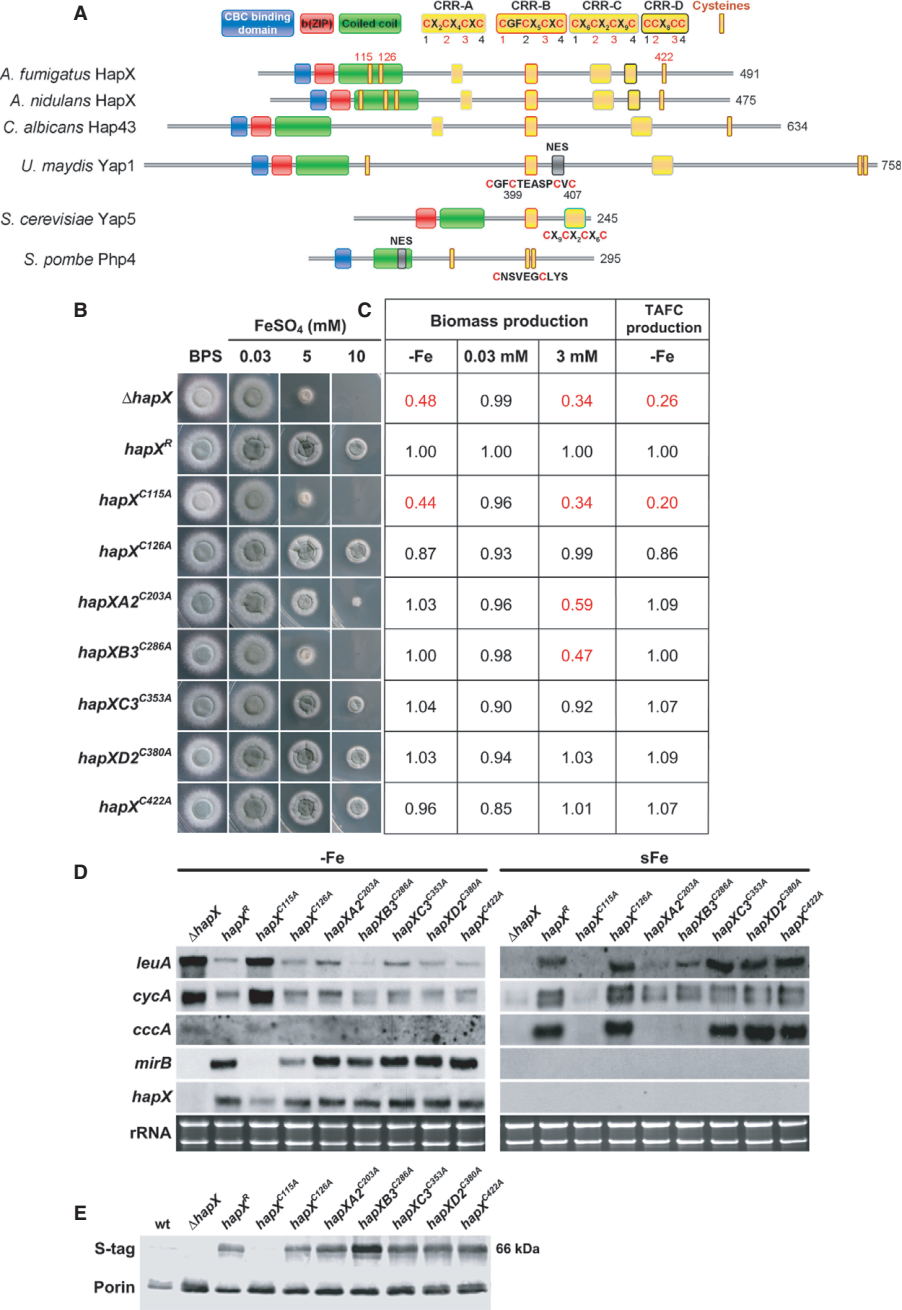
CRR-B and, to a lesser degree, CRR-A are crucial for iron detoxification but not adaptation to iron starvation Schematic view of the HapX Cys and domain organization including comparisons of HapX orthologs from *A. fumigatus*, *A. nidulans*, *C. albicans*, *S. pombe, Ustilago maydis* Yap1, and *S. cerevisiae* Yap5.Strains were grown for 48 h at 37°C on agar plates with the given iron concentration.Production of biomass during iron starvation (−Fe), iron sufficiency (0.03 mM, +Fe), and iron excess (3 mM, hFe), as well as production of siderophores under iron starvation was monitored after liquid growth for 24 h at 37°C. The data represent the mean ± SD of biological triplicates; the values were normalized to that of strain *hapX*^*R*^ carrying a non-mutated S-tagged *hapX*. Statistically significant differences compared to *hapX*^*R*^ are shown in red (two-tailed, unpaired *t*-test; *P* < 0.05). Original data with standard deviations are given in Supplementary Table S1.Northern blot analyses were performed after liquid growth for 24 h at 37°C under iron limitation (−Fe) or after a subsequent 1-h shift into iron sufficiency (sFe). rRNA is shown as a control for RNA quantity and quality.Western blot analyses were performed after liquid growth for 24 h at 37°C under iron limitation using antisera recognizing the S-tag for detection of HapX, or porin as control for loading. We were unable to detect S-tagged HapX during iron sufficiency or high-iron conditions with this method (data not shown). Data information: For simplicity, only one mutant per CRR is shown, the respective, phenotypically identical second mutant is shown in Supplementary Fig S3. Strains are derivatives of *A. fumigatus* AfS77.

To analyze the expression and localization of *A. fumigatus* HapX at the protein level, we generated an *A. fumigatus* strain expressing HapX N-terminally tagged with the Venus fluorescent protein (a derivative of yellow fluorescent protein) (Nagai *et al*, 2002), under the control of the endogenous *hapX* promoter in single copy at the *hapX* locus in Δ*hapX* (strain *hapX*^*VENUS*^). This cured all mutant phenotypes on solid and in liquid media, indicating that the HapX^VENUS^ protein is fully functional (Supplementary Table S1). In agreement with the transcriptional data, in epifluorescence microscopy HapX^VENUS^ was detectable during iron starvation but not during iron sufficiency, iron excess or a 1-h shift from iron starvation to iron sufficiency (Fig3B). As previously observed in *A. nidulans* and *S. pombe* (Hortschansky *et al*, 2007; Mercier & Labbe, 2009), *A. fumigatus* HapX^VENUS^ localized to the nucleus during iron starvation. These data indicate that lower protein levels of HapX are required for its functions during iron excess compared to iron starvation. Consistently, S-tagged HapX (strain *hapX*^*R*^) was detectable only during iron starvation (Fig4E) but not during iron excess (data not shown) in Western blot analyses. These data also demonstrate that expression pattern-based prediction of gene functions can be misleading. In order to increase the sensitivity of HapX protein detection, we enriched HapX^VENUS^ by GFP-trap, a commercially available GFP pull-down (Rothbauer *et al*, 2008), before Western blot analysis with a GFP-directed antiserum was applied. This way, the HapX^VENUS^ protein was detected in mycelia grown under iron starvation, iron-replete as well as high-iron conditions with the lowest amount present under high-iron conditions (Fig3C). Under iron starvation, significant HapX proteolyses was found. Most likely, the Venus-HapX degradation was caused during the non-denaturating GFP enrichment procedure. The highly increased degradation during iron starvation conditions can be explained by the strong induction of protease activity during iron starvation conditions (data not shown and Supplementary Table S2). However, it cannot be ruled out that this proteolysis reflects a higher HapX turnover during iron starvation, which might be related to the increased transcript level under this condition. The reduced HapX protein content during iron excess compared to iron starvation might be explained by the reduced number of target genes expressed under this condition.

### Two cysteine-rich regions (CRR), CRR-A and CRR-B, are crucial for HapX-mediated iron resistance

*Aspergillus fumigatus* HapX, 491 amino acid residues in length, contains the following domains: a “b(ZIP)” basic and a “coiled-coil” domain, which together mediate DNA-binding in bZIP-type transcription factors, and an N-terminal CBC-binding domain that is essential for HapX function due to its requirement for interaction with the CBC subunit HapE (Hortschansky *et al*, 2007). Moreover, HapX harbors the enormous number of 19 cysteine residues (Cys), whereby 16 are organized in 4 clusters, termed CRR-A, CRR-B, CRR-C, and CRR-D containing four Cys each (Fig4). Two single Cys (Cys115 and Cys126) are localized in the coiled-coil region, and another one (Cys422) is localized in the C-terminus. The importance of these Cys is supported by their evolutionary conservation, for example all Cys are conserved in seven *Aspergillus* species (Supplementary Fig S1); CRR-A, CRR-B, CRR-C as well as the C-terminal Cys are conserved even in distantly related fungal species such as *C. albicans* (Fig4A and Supplementary Fig S2).

Due to the potential role of Cys in iron sensing (Lill *et al*, 2012), we studied the impact of 11 of these Cys on HapX functions by site-directed mutagenesis replacing Cys by alanine residues (Fig 4 and Supplementary Fig S3). This analysis included all three single as well as two Cys from each CRR. For simplicity, only one mutant per CRR is shown in Fig 4, the respective, phenotypically identical second mutant is shown in Supplementary Fig S3. All analyzed *hapX* versions, including the non-mutated (strain *hapX*^*R*^), were expressed under the control of the endogenous promoter, contained a C-terminal S-tag (Terpe, 2003) and were integrated at the *hapX* locus in the Δ*hapX* strain.

Mutations in CRR-B (strains *hapXB1*^*C277A*^ and *hapXB3*^*C286A*^) dramatically decreased adaptation to iron excess, similar to HapX-deficiency (Δ*hapX*), reflected by decreased radial growth under high-iron conditions, decreased biomass production in high-iron media as well as impaired transcriptional induction of *cccA* and *leuA* during a shift from iron starvation to iron sufficiency (Fig 4 and Supplementary Fig S3). Compared to CRR-B mutations, CRR-A mutations (strains *hapXA2*^*C203A*^ and *hapXA3*^*C208A*^) similarly impaired the transcriptional response of *cccA* and *leuA* to iron, but the growth of the mutant strain was significantly increased on solid as well as in liquid high-iron media (Fig 4 and Supplementary Fig S3). Mutations in CRR-C (strains *hapXC2*^*C350A*^ and *hapXC3*^*C353A*^) led to a slightly decreased growth on solid and in liquid high-iron media, but did not affect the transcription pattern of *cccA* and *leuA* (Fig 4 and Supplementary Fig S3). Notably, mutation of two different Cys in the same CRR impaired iron resistance to the same degree in CRR-A, CRR-B, and CRR-C (Fig 4 and Supplementary Fig S3) suggesting that the Cys in the same CRR act as a domain rather than individually. Remarkably, neither of the mutations in CRR-A, CRR-B and CRR-C did affect the growth during iron sufficiency or limitation (Fig4). Consistently, siderophore production and transcript levels of HapX-repressed genes (*cccA, leuA, cycA*) as well as HapX-activated *mirB* were wild-type-like (strain *hapX*^*R*^) in all these mutants under iron limitation (Fig4). Taken together, these data demonstrate that mainly CRR-B, but to a lower degree also CRR-A and even less CRR-C are required for the HapX functions in iron detoxification, while these CRR are dispensable for the HapX functions in adaptation to iron starvation.

Mutation of Cys126 (strain *hapX*^*C126A*^) slightly decreased liquid biomass production and TAFC production during iron starvation but did not impact HapX functions in any other assays performed (Fig4). Mutation of Cys422 (strain *hapX*^*C422A*^) slightly decreased liquid biomass production under both iron starvation and excess but did not cause any other alterations (Fig4). Mutations in CRR-D (strains *hapXD2*^*C380A*^ and *hapXD3*^*C389A*^) were phenotypically inconspicuous in all analyses performed (Fig4).

In contrast, mutation of the Cys115 (strain *hapX*^*C115A*^) phenocopied HapX-deficiency during both iron limitation and iron excess (Fig4). The most likely explanation is that this mutation results in the loss of the HapX protein as seen in Fig4E, possibly due to improper folding of this HapX derivative. Noteworthy, this mutation results in a decrease of *hapX* transcript level during iron starvation suggesting positive transcriptional autoregulation of HapX.

### The HapX C-terminus is essential for the adaption to iron starvation

**Figure 5:**
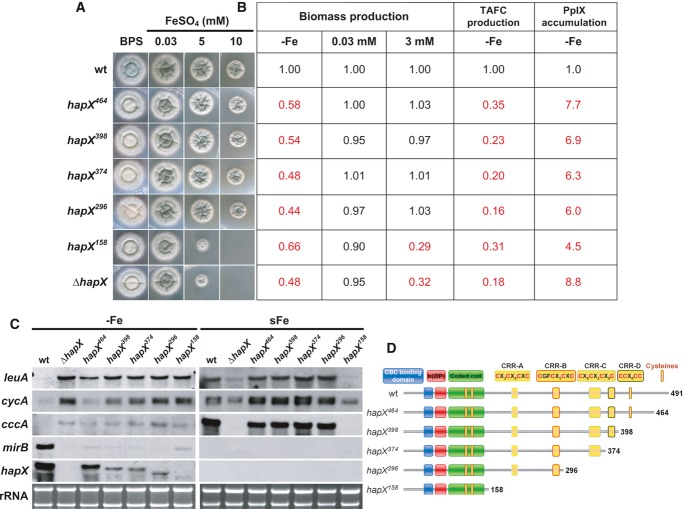
The HapX C-terminus is crucial for adaption to iron starvation but not iron detoxification Strains were grown for 48 h at 37°C on agar plates with the given iron concentration.Production of biomass during iron starvation (−Fe), iron sufficiency (0.03 mM, +Fe), and iron excess (3 mM, hFe), as well as production of siderophores and PpIX under iron starvation was monitored after liquid growth for 24 h at 37°C. The data represent the mean ± SD of biological triplicates; the values were normalized to the wild-type. Statistically significant differences compared to the wild-type are shown in red (two-tailed, unpaired *t*-test; *P* < 0.05). Original data ± SD are found in Supplementary Table S1.Northern blot analyses were performed after liquid growth for 24 h at 37°C under iron limitation (−Fe) or after a subsequent 1-h shift to iron sufficiency (sFe). rRNA is shown as a control for RNA quantity and quality.Schematic view of the HapX truncations analyzed. Data information: Strains are derivatives of *A. fumigatus* AfS77.

To further characterize HapX domains, we generated *A. fumigatus* strains expressing different C-terminal *hapX* truncations (strains *hapX*^*464*^ – *hapX*^*158*^), here untagged, under the control of the endogenous promoter and integrated at the *hapX* locus in Δ*hapX* (Fig5D).

Truncation of the C-terminal 27 amino acid residues (strain *hapX*^*464*^*)* decreased sporulation on BPS agar plates (Fig5A). In iron-limited liquid media, this truncation decreased biomass production, TAFC production, *mirB* transcript levels but increased protoporphyrine IX (PpIX) accumulation and transcript levels of *cccA* and *leuA* (Fig5). Taken together, this truncation was similar to a Δ*hapX* phenotype during iron starvation, however, less severe (with regard to biomass and TAFC production as well as the *cycA* transcript level), but did not affect growth and transcription of iron-related genes under iron excess, i.e. *cccA* and *leuA* (Fig5).

Truncation of the C-terminal 93, 117, and 195 amino acid residues (strains *hapX*^*389*^, *hapX*^*374*^ and *hapX*^*296*^, respectively) perfectly phenocopied Δ*hapX* under iron limitation but did not affect iron detoxification (Fig5). The latter is consistent with the presence of CRR-A and CRR-B that are crucial for iron resistance in the respective HapX versions (see above). The functionality of the C-terminus is supported by the high evolutionary conservation not only in *Aspergillus* species (Supplementary Fig S1) but also for example in *C. albicans* with 41% identical amino acids in the C-terminal 66 amino acid residues (data not shown).

Truncation of the C-terminal 333 amino acid residues (strain *hapX*^*158*^) impaired iron detoxification to the same extent as HapX-deficiency, which is in agreement with the lack of CRR-A and CRR-B (Fig5). This truncation also reduced adaptation to iron starvation, but not to the same extent as seen in *hapX*^*464*^ – *hapX*^*296*^ or Δ*hapX,* i.e. during iron limitation liquid biomass and TAFC production as well as the *mirB* transcript level were higher, while the PpIX accumulation was lower. These data indicate that this HapX version, comprised of only the CBC-binding domain and the bZIP region, still executes residual functions in activation of siderophore biosynthesis and repression of iron consumption. These functions appear to be masked in the HapX versions encoded by *hapX*^*464*^ – *hapX*^*296*^.

Notably, *hapX*^*398*^ – *hapX*^*158*^ displayed not only decreased transcriptional activation of *mirB* but also decreased *hapX* transcript levels as seen in the HapX loss of function *hapX*^*C115A*^ mutant (see above). These data indicate positive transcriptional autoregulation of HapX.

Taken together, both the cysteine-to-alanine mutations and the C-terminal truncations indicate that CRR-A and CRR-B are required for HapX-mediated iron detoxification, while the C-terminal 93 amino acid residues are crucial for both activation as well as repression functions that are required for adaptation to iron starvation.

### An evolutionary conserved *cccA* promoter element is recognized by a protein complex consisting of the CBC and a HapX homodimer involving direct DNA binding by both the CBC and HapX

**Figure 6:**
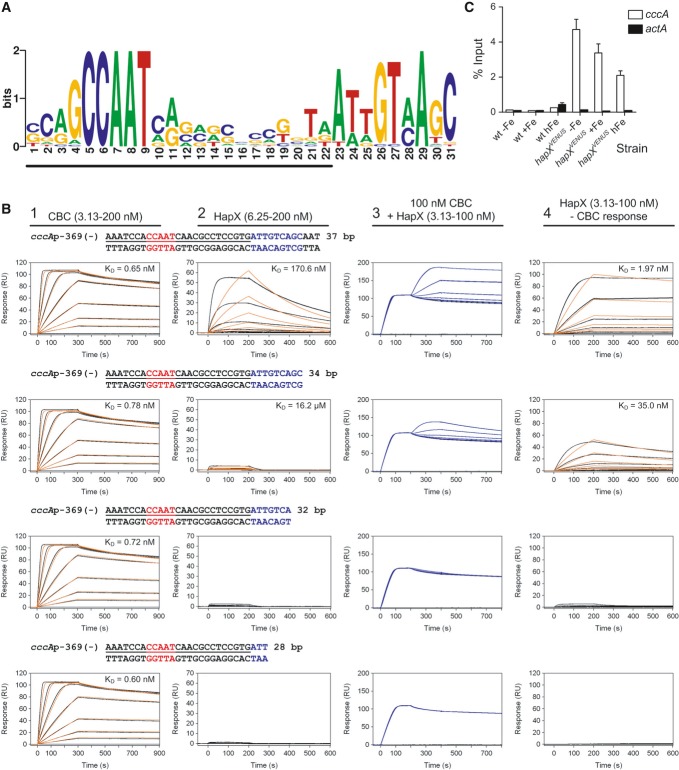
HapX binds *in vitro* and *in vivo* to an evolutionary conserved motif identified in promoters of *cccA* homologs An evolutionary conserved, bipartite motif in promoters of *cccA* homologs identified by MEME analysis. The underlined nucleotides would be covered upon CBC-binding as can be predicted based on the identified CBC/DNA binary complex crystal structure (Huber *et al*, 2012).Real-time SPR characterization of *in vitro* formation of the CBC/DNA-HapX ternary complex on the conserved *cccA* promoter motif from *A. fumigatus*. SPR analyses included binding of the CBC to DNA (panel 1), HapX to DNA (panel 2) and HapX to preformed CBC/DNA complexes (panel 3). The SPR sensorgrams are shown from sensor-immobilized 37 base pair duplexes covering the full as well as 3′-truncated duplexes. Nucleotides marked in blue represent the HapX consensus binding site in fungal *cccA* promoters identified by MEME analysis. Binding responses of the indicated CBC or HapX concentrations injected in duplicate (black lines) are shown overlaid with the best fit derived from a 1:1 interaction model including a mass transport term (red lines). Binding responses of CBC/DNA-HapX ternary complex formation (panel 3, blue lines) were obtained by concentration-dependent co-injection of HapX on preformed binary CBC/DNA complexes after 200 s within the steady-state phase. Sensorgrams in panel 4 depict the association/dissociation responses of HapX on preformed CBC/DNA and were generated by CBC response (co-injection of buffer instead of HapX) subtraction from HapX co-injection responses. Dissociation constants (*K*_D_) are plotted inside the graphs.ChIP analysis demonstrating *in vivo* binding of HapX to the conserved *cccA* promoter motif from *A. fumigatus*. ChIP qPCR was performed on wild-type or the strain containing Venus-tagged HapX (*hapX*^*VENUS*^) grown for 18 h, then shifted to fresh media with no iron (−Fe), 0.03 mM iron (+Fe), or 3 mM iron (hFe) for 8 h. DNA was immunoprecipitated with either a control IgG antibody, or anti-GFP polyclonal antibody that recognizes the Venus protein. Binding of HapX^VENUS^ to the DNA region was assessed by qPCR. HapX binding is represented as percent enrichment of input control samples ± SD from triplicates. The *actA* (actin) promoter served as a negative control.

To identify putative, evolutionary conserved, regulatory motifs in the *cccA* promoter, the 1-kb 5′-upstream regions of *cccA* homologs from 28 fungal species including *A. fumigatus, A. nidulans* and *F. oxysporum* were subject to MEME analysis (Bailey & Elkan, 1994). The identified sites and their positions in the promoters of the different species are shown in Supplementary Fig S4. The highest scoring sequence (*e*-value of 3.4 × 10^−115^), present in all 28 species, was a bipartite motif separated by a spacer region with low conservation (Fig6A). Consistent with the HapX-independent regulation, the highest scoring motif was not found in the promoter of the *S. cerevisiae cccA* homolog (data not shown). The 5′-conserved submotif matches the CBC consensus DNA-binding motif (CCAAT box), CCAAT(C/T)(A/G) (Huber *et al*, 2012). This is in perfect agreement with the previous finding that HapX acts via physical interaction with the CBC (Hortschansky *et al*, 2007). Interestingly, binding of the CCAAT box by CBC would cover the entire spacer region as identified in the CBC/DNA binary complex crystal structure (Huber *et al*, 2012), which indicates that the 3′-submotif is the first accessible region for binding of another DNA-binding protein. The 3′-conserved non-palindromic submotif does not match any known transcription factor consensus binding sequence. This is intriguing, because bZIP proteins usually bind short palindromic or pseudo-palindromic target sequences. Furthermore, based on the amino acid signature sequence of its basic region **N**XX**AQ**XX**FR** (Supplementary Fig S1), HapX belongs to the Pap1/Yap1 subfamily of bZIP transcription factors that are known to recognize **TTA**CG**TAA** and **TTA**G**TAA** consensus motifs (Fujii *et al*, 2000).

Nevertheless, we postulated that the 3′-submotif might be recognized by HapX. To address this hypothesis, we overexpressed and purified the *A. fumigatus* CBC (comprising the conserved domains of subunits HapB, HapC, and HapE) as well as a peptide corresponding to residues 24–158 of HapX, which includes the CBC-binding domain, basic region, and coiled-coil domain (Supplementary Fig S5A). Light scattering analysis of purified HapX (24–158) revealed a molar mass of 31.38 kDa, demonstrating that this domain is dimeric in solution (theoretical mass of 31.36 kDa), as expected for a bZIP protein (Supplementary Fig S5B). To examine the putative *in vitro* interaction of the CBC, HapX and the identified common promoter element of *cccA* homologs, we applied surface plasmon resonance (SPR) analyses (Fig6B). These measurements indicated high-affinity (*K*_D_ = 0.7 nM) recognition of the CCAAT box by the *A. fumigatus* CBC independent of the presence of the 3′-submotif, i.e. its binding affinity was not affected by truncation of the 3′-submotif (compare first column in Fig6B). This affinity is similar to that found for the interaction of the *A. nidulans* CBC with a CCAAT motif from the *sreA* promoter (Thon *et al*, 2010). HapX binds the 3′-submotif with low affinity (*K*_D_ = 170.6 nM) as its binding was abolished by truncation of the 3′-submotif (compare second column in Fig6B). However, on CBC-coated DNA, the binding affinity of HapX was 87-fold increased (*K*_D_ ≈ 1.97 nM), whereby the binding again strictly depended on the presence of the 3′-submotif (compare fourth column in Fig6B). Furthermore, by taking advantage of the fact that SPR responses correspond to bound masses, we were able to unravel the stoichiometry of the CBC/DNA-HapX ternary complex. Saturating CBC responses on the 37-bp DNA duplex reached a value of ≈ 100 RU (upper left sensorgram in Fig6B) and nearly the same responses were observed by co-injection of HapX on preformed CBC/DNA complexes (upper right sensorgram in Fig6B) due to the similar molecular masses of the CBC (33.44 kDa) and the HapX dimer (31.36 kDa). Therefore, we conclude that one binary CBC/DNA complex is bound by one HapX dimer. Taken together, our data demonstrate that HapX interacts *in vitro* not only with the CBC but also with DNA with 2:1:1 stoichiometry by recognizing the 3′-submotif located adjacent to the CCAAT box in the evolutionary conserved *cccA* promoter element.

Chromatin immunoprecipitation (ChIP) analysis confirmed that HapX also binds to this promoter element *in vivo*, notably independent of the ambient iron concentration (Fig6C). The constitutive presence of HapX at the *cccA* promoter suggests that transcription of *cccA* is primarily mediated by post-translational modification of HapX, i.e. iron sensing, rather than by transcriptional regulation of *hapX*. In agreement, the potential HapX iron-sensing CRR-B motif, **C**GF**C**X_5_**C**X**C**, is essential for the transcriptional activation of *cccA* in response to high-iron stress (see above).

In summary, we show that HapX not only physically interacts with the CBC but also directly recognizes a distinct DNA motif. As the CBC has numerous HapX/iron-independent functions (Kato, 2005; Fleming *et al*, 2013), these data reveal for the first time the mechanism for discrimination of general CBC and specific HapX/CBC target genes.

### Both functions, adaptation to iron limitation and excess, are evolutionary conserved in HapX orthologs

**Figure 7:**
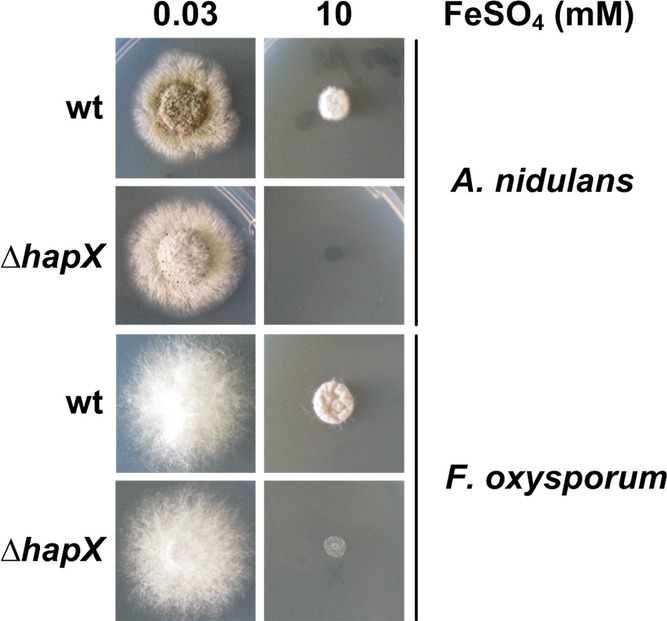
HapX-mediated iron detoxification is evolutionary conserved *A. nidulans* and *F. oxysporum* deletion mutants were grown for 48 h at 37°C on agar plates with the given iron concentration.

Similar to *A. fumigatus*, HapX orthologs repress iron-dependent pathways and the *cccA* orthologs during iron starvation in *A. nidulans*, *F. oxysporum, S. pombe, C. neoformans,* and *C. albicans* (Mercier *et al*, 2006; Hortschansky *et al*, 2007; Jung *et al*, 2010; Schrettl *et al*, 2010; Hsu *et al*, 2011; Lopez-Berges *et al*, 2012). Here, we found that HapX-deficiency also impairs growth of *A. nidulans* and *F. oxysporum* on high-iron media (Fig7) demonstrating that the function of HapX in iron detoxification is evolutionary conserved. Inspection of genome-wide transcriptional profiling data indicated that the *cccA* ortholog FOXG_04047 in *F. oxysporum* is repressed similar to *A. fumigatus* during iron starvation and upregulated during iron sufficiency in a HapX-dependent manner (Lopez-Berges *et al*, 2012). These data suggest that in *F. oxysporum* the decreased iron resistance caused by HapX-deficiency also results from impaired vacuolar iron storage.

In agreement with the evolutionary conserved function in iron resistance, CRR-A and CRR-B, which were identified in this study to be crucial for transcriptional activation of *cccA* and consequently iron detoxification in *A. fumigatus,* are conserved in most HapX orthologs (Fig4A and Supplementary Fig S2).

In *S. cerevisiae*, the *cccA* ortholog, *ccc1*, is post-transcriptionally repressed during iron starvation by Cth1/2-mediated mRNA decay (Puig *et al*, 2005), which is transcriptionally induced by Aft1. Transcriptional activation during iron excess is mediated by the bZIP-type transcription factor Yap5. *S. cerevisiae* lacks HapX and SreA orthologs and, vice versa, *A. fumigatus* lacks orthologs of Aft1, Cth1/2, and Yap5 (Haas *et al*, 2008). Like *A. fumigatus* HapX, *S. cerevisiae* Yap5 comprises a bZIP domain. However, Yap5 lacks a CBC-binding domain, does not function via interaction with the CBC, and is involved in adaptation to iron excess but not iron starvation (Li *et al*, 2008). Thus, transcriptional activation of vacuolar iron storage is mediated by different regulatory mechanisms in *S. cerevisiae* and *A. fumigatus*. Nevertheless, it is particularly interesting that CRR-B, which is mainly important for the function of HapX in iron resistance, shows significant similarity to a CRR that is likewise essential for the iron resistance function of *S. cerevisiae* Yap5 (Fig4A).

The common CRR-B motif, **C**GF**C**X_5_**C**X**C**, which is essential for the function in iron resistance of HapX and Yap5, is reminiscent of the CGFS motif found in monothiol glutaredoxins. Here, this motif is essential for the formation of [2Fe-2S]-bridged homodimers (Picciocchi *et al*, 2007; Bandyopadhyay *et al*, 2008; Iwema *et al*, 2009) that participate in mitochondrial and cytosolic iron–sulfur protein biogenesis (Rouhier *et al*, 2010), delivery and transfer of iron–sulfur clusters into proteins and subcellular compartments (Muhlenhoff *et al*, 2010), and the relay of the cellular iron status to iron-responsive transcription factors (Rutherford *et al*, 2005; Ojeda *et al*, 2006; Pujol-Carrion *et al*, 2006; Kaplan & Kaplan, 2009). Consistent with a role of CRR-B in sensing the cellular iron status via iron–sulfur clusters in *S. cerevisiae* Yap5, and potentially *A. fumigatus* HapX, inactivation of iron–sulfur cluster biosynthesis blocks Yap5 activation in yeast (Li *et al*, 2012). Moreover, the second CRR present in Yap5 shows similarity to CRR-C of HapX (Fig4A).

Php4, which transcriptionally represses iron-dependent pathways in *S. pombe* (Vachon *et al*, 2012), is an atypical HapX ortholog comprising a CBC-binding domain and a coiled-coil domain, but lacking the basic DNA-binding region of the bZIP domain. Only three Cys are present with two being clustered in the sequence **C**NSVEG**C**LYS (Fig4A). In a two-hybrid approach, the first of these Cys, Cys172, was found to be essential for iron-dependent association of Php4 with the monothiol glutaredoxin Grx4, indicating a role in iron sensing via iron–sulfur cluster availability. However, the function of this Cys has not been analyzed directly in Php4.

Recently, sulfur starvation caused by deficiency in the sulfur regulator MetR was found to increase the cellular iron content, iron sensitivity and transcript levels of genes involved in iron uptake but to decreased the *cccA* transcript level (Amich *et al*, 2013). These data demonstrate that sulfur homeostasis is required for proper iron regulation, most likely via iron-sulfur cluster and/or glutathione biosynthesis as shown for *S. cerevisiae* and *S. pombe* (Li & Outten, 2012). Together with the results presented here, these data furthermore indicate that sulfur homeostasis is required not only for SreA-mediated regulation of iron uptake but also HapX-mediated regulation of vacuolar iron detoxification.

Taken together, CRR-A and -B, but not the C-terminus, are required for sensing of high-iron conditions by HapX to mediate activation of vacuolar iron detoxification via a conformational change of and/or interaction with different protein partners compared to iron starvation conditions. In contrast, the C-terminal 93 amino acid residues, but not CRR-A and CRR-B, are crucial for both the activating (e.g. *mirB*) as well as repressing (e.g. *cccA*) functions of HapX during iron starvation. The intriguing question why some promoter interactions result in repression and some in activation has still to be resolved; again this might be dependent on interaction with different partners present in different promoter sets.

Strikingly, certain domains of HapX and Yap5 are also found in the *Ustilago maydis* peroxide sensor, named Yap1 (Molina & Kahmann, 2007). This protein was identified as an ortholog of Yap1 from *S. cerevisiae* and regulates the oxidative stress response of the plant pathogen. A closer inspection of the Yap1 amino acid sequence revealed the presence of common HapX domains, i.e. the N-terminal CBC-binding and bZIP domains, a central HapX/Yap5 CRR-B type motif and the C-terminal CRR-C domain. Two additional Cys (CPC-motif) are located close to the C-terminus, whereas HapX-like domains CRR-A and CRR-D are lacking. Within the CRR-B domain, Cys399, and Cys407 were found to be crucial for nuclear localization and function of *U. maydis* Yap1, probably due to oxidative masking of a putative nuclear export sequence located between CRR-B and CRR-C (Fig4A and Supplementary Fig S2) (Delaunay *et al*, 2000). Taken together, it seems that different fungi use a toolbox-like set of domains for sensing distinct environmental stimuli. Furthermore, these data indicate a possible role of Yap1 as a Yap1/HapX chimera in regulation of *U. maydis'* iron homeostasis.

## Conclusion

Collectively, our study uncovered a novel regulatory mechanism in *A. fumigatus* and other filamentous fungi mediating both iron resistance and adaptation to iron starvation by the same transcription factor complex, comprising the CBC and a HapX homodimer, with activating and repressing functions depending on ambient iron availability. Comparison of HapX target promoter regions coupled with protein-DNA interaction analysis identified an evolutionary conserved CBC/HapX binding motif and revealed the discriminatory mechanism for CBC and CBC/HapX targets. Moreover, mutational analysis identified HapX protein domains that are essential for adaptation to either limitation or excess of iron, which will be instrumental for the further characterization of iron sensing.

Most fungal species encode HapX orthologs indicating wide evolutionary conservation of this iron regulatory mechanism. In contrast, the role model *S. cerevisiae* lacks a HapX ortholog and employs iron regulatory transcription factors that are not found in most other fungal species (Haas *et al*, 2008; Kaplan & Kaplan, 2009). In addition to the high-iron-sensing Yap5, iron regulation in *S. cerevisiae* involves the low-iron-sensing paralogous transcription factors Aft1 and Aft2. Aft1 and Aft2 transcriptionally activate iron acquisition as well as Cth1 and Cth2. These two paralogous mRNA-binding proteins mediate post-transcriptional repression of iron consumption by promoting respective mRNA degradation, including the homolog of *A. fumigatus cccA* (Martinez-Pastor *et al*, 2013). These data demonstrate complete regulatory rewiring of vacuolar iron storage in this yeast compared to *A. fumigatus,* with different regulators being required for activation and repression. Nevertheless, the mechanistically different iron regulatory systems of *A. fumigatus* and *S. cerevisiae* seem to employ common protein motifs for mediation of iron regulation. As an example of the modular toolbox using a common protein motif for transmitting different signals, the N-terminal CBC-binding domain of HapX is also present in *S. cerevisiae* Hap4. Hap4 was recently suggested to participate in iron regulation (Ihrig *et al*, 2010). In contrast to HapX, however, Hap4 lacks DNA binding and CRRs and therefore its mode of action is significantly different from the HapX mechanism (McNabb & Pinto, 2005; Hortschansky *et al*, 2007). In mammals, iron acquisition (transferrin receptor) and iron detoxification (ferroportin-mediated iron export and ferritin-mediated iron storage) are coupled at the post-transcriptional level by responding inversely to binding of iron regulatory proteins (IRPs) to iron-responsive elements in the untranslated regions of respective mRNA′s (Wang & Pantopoulos, 2011).

Taken together, the comparison of different organisms underlines the essentiality of iron handling with similar readouts mediated by different regulatory mechanisms.

## Materials and Methods

### Strains, oligonucleotides, and growth conditions

All strains and oligonucleotides used in this study are listed in Supplementary Tables S3 and S4, respectively. Generally, *A. fumigatus* strains were cultivated at 37°C in *Aspergillus* minimal medium (AMM) according to Pontecorvo *et al* (1953) containing 1% (w/v) glucose and 20 mM glutamine as carbon and nitrogen sources, respectively. For iron-depleted conditions, iron was omitted and iron amounts used in this study are given in the figures. To increase iron starvation in solid media, the ferrous iron chelator BPS was used at a final concentration of 0.2 mM in iron-depleted media. Production of conidia was performed on AMM agar plates containing 0.03 mM FeSO_4_. Depending on the transformed resistance gene, *A. fumigatus* strains were selected on media containing 0.1 μg/ml pyrithiamine or 0.2 mg/ml hygromycin B.

### Generation of a *hapX* mutant strain with conditional *cccA* expression

For heterologous expression of *cccA*, the plasmid pxylP^P^cccA (Gsaller *et al*, 2012) was transformed in *A. fumigatus* Δ*hapX* (ATCC 46645) (Supplementary Fig S6). The plasmid harbors *cccA* under control of *xylP*^*P*^, a xylan/xylose-inducible promoter derived from the xylanase *xylP* of *Penicillium chrysogenum* (Zadra *et al*, 2000). pAN7.1 containing a hygromycin resistance cassette (*hph*) was co-transformed for selection of transformants.

### Generation of a *hapX* deletion mutant in Δ*akuA* background and a Δ*hapX*Δ*cccA* double deletion strain

The *hapX* coding sequence was deleted in AfS77 using the bipartite marker technique (Supplementary Fig S7A) (Nielsen *et al*, 2006). AfS77 was transformed with two DNA fragments each containing overlapping but incomplete fragments of the pyrithiamine resistance-conferring gene *ptrA* as described previously (Schrettl *et al*, 2010) yielding strain Δ*hapX* (Supplementary Fig S7A). Deletion constructs were amplified with primers oAfhapX-5/oAoPtrA2 (PCR1, 2.6 kb) and oAfhapX-6/oAoPtrA1 (PCR2, 2.2 kb) using genomic DNA of Δ*hapX* (ATCC 46645 derivative) as template.

Disruption of *cccA* in Δ*hapX* background (AfS77) was also carried out using the bipartite marker technique (Supplementary Fig S7B). For this purpose, the *cccA* 5′-flanking region was amplified from genomic DNA using primers oAfcccA1/oAfcccAr4. For amplification of the 3′-flanking region, primers oAfcccA3/oAfcccAr2 were employed. Generated DNA fragments were digested with *Stu*I (5′-flanking region) and *Hind*III (3′-flanking region). The hygromycin resistance cassette was released from plasmid pAN7.1 by digestion with *Stu*I and *Hind*III and ligated with the 5′- and 3′-flanking region, respectively. The transformation construct A (3.3 kb, fusion of the *cccA* 5′-flanking region and the *hph* split marker) was amplified from the ligation product using primers oAfcccA5 and ohph14. For amplification of the transformation construct B (2.8 kb, fusion of the *cccA* 3′-flanking region and the supplementary *hph* split marker) primers oAfcccAr6 and ohph15 were employed. For transformation of *A. fumigatus* strains, both constructs A and B were used simultaneously. Δ*hapX* (AfS77) was transformed with the fragments each containing overlapping but incomplete fragments of the hygromycin resistance gene *hph*, yielding strain Δ*hapX*Δ*cccA*.

### *ΔhapX* complementation, site-directed mutagenesis, C-terminal truncation, S-tagging, and venus-tagging of HapX

For these studies*,* the basic plasmid phapX^R^-hph was generated employing the fusion PCR-technique (Nielsen *et al*, 2006). The resulting plasmid contains the *hapX* coding sequence C-terminally linked with an S-tag under control of its native promoter and terminator region as well as a hygromycin resistance cassette. The different plasmid versions were integrated at the *hapX* deletion locus in AfS77 Δ*hapX* (Supplementary Fig S8A).

As a first step, the sequence encoding the S-tag (72 bp), including a 5′-sequence coding for a GA_4_-linker and a 3′-stop codon (TAA), was PCR amplified from plasmid pAO81 (Yang *et al*, 2004) using primers oAfhapX-S1 and oAfhapX-S2 (Supplementary Table S4). These primers carry extensions, which are complementary to sequences 30 bp upstream (oAfhapX-S1) and downstream (oAfhapX-S2) of the *hapX* stop codon (TGA), thereby generating a 132-bp-long PCR product. Primers oAfhapX-1 and oAfhapX-S3 were employed to amplify 2.9 kb of genomic DNA sequence, comprising 5′-flanking region (including the *hapX* promoter) and the *hapX* gene lacking the endogenous stop codon (TGA). Primers oAfhapX-S2 and oAfhapX-S4 were used to amplify 1.0 kb of genomic sequence, comprising 3′-flanking region (*hapX* terminator region). After gel purification, equal molar amounts of the PCR products were applied as template for fusion PCR. By using the (nested) primers oAfhapX-7 and oAfhapX-8 a 3.7 kb long fragment was amplified, which contains the *hapX* promoter, the *hapX* coding sequence comprising the S-tag, and the *hapX* terminator region. Subsequently, 3′ A-overhangs were added and the construct was subcloned into pGEM-T-Easy (Promega) via T/A cloning, yielding phapX^R^ (6.7 kb). Finally, a hygromycin resistance cassette was subcloned in phapX^R^, yielding plasmid phapX^R^-hph. Therefore, a DNA fragment containing the resistance cassette (2.4 kb) was amplified with primers ohyg-1/ohyg-2 using pAN7.1 as template. Next, phapX^R^ was opened with *Sph*I and blunt-ended using the Klenow enzyme (NEB). Eventually, the amplified DNA fragment was phosphorylated with polynucleotide kinase (NEB) and ligated into the blunt-ended plasmid backbone.

In order to substitute specific amino acids by site-directed mutagenesis the QuikChange kit (Stratagene) and oligonucleotides listed in Supplementary Table S4 were used. For the introduction of each mutation (Fig4B and Supplementary Fig S3), complementary primers, around 40 bp in length including the desired mutation in its center, were designed. Plasmid phapX^R^-hph was amplified with respective primers in a PCR with a total volume of 50 μl (18 cycles, *T*_Den_ = 95°C, *T*_Melt_ = 56°C, *T*_Elong_ = 72°C). Next, 1 μl *Dpn*I restriction enzyme was added to the solution followed by 3 h of incubation at 37°C. *E. coli* DH5α cells were transformed with 10 μl of the digested solution. After amplification of plasmid DNA, plasmids containing mutated coding sequences were screened by digestion. Resulting plasmids were named phapX^C115A^-hph, phapX^C126A^-hph, phapXA2^C203A^-hph, phapXA3^C208A^-hph, phapXB1^C277A^-hph, phapXB3^C286A^-hph, phapXC2^C350A^-hph, phapXC3^C353A^-hph, phapXD2^C380A^-hph, phapXD3^C389A^-hph, and phapX^C422A^-hph.

For C-terminal truncation of HapX, reverse primers were designed that anneal to the *hapX* coding sequence at specific sites (Supplementary Table S4). Additionally, reverse primers comprise a STOP codon and a *Bst*BI recognition site. Each PCR was performed with the same forward primer – hapXtrunc-f. Amino acid lengths of truncated HapX proteins (full-length in Af293 = 491 aa) are listed in Supplementary Table S3 and Fig5D. After amplification, PCR fragments were digested with *Xba*I/*Bst*BI and subcloned into *Xba*I/*Bst*BI opened phapX^R^-hph. Plasmids comprising the truncated *hapX* versions were designated phapX^464^-hph, phapX^398^-hph, phapX^374^-hph, phapX^296^-hph, and phapX^158^-hph.

For N-terminal tagging of HapX with Venus fluorescent protein plasmid phapX^VENUS^-hph was generated (Supplementary Fig S8B). Therefore, *hapX* promoter DNA was amplified using primers 5′hapX-f/5′hapXvenus-r (PCR1, 1.3 kb; template: phapX^R^-hph) (Supplementary Table S4). Primers 5′hapXvenus-f/venus-r were employed to amplify the codon optimized *venus* coding sequence (PCR2, 0.7 kb; template: pMA-Venus). In a third PCR, *hapX* coding sequence comprising the 3′ untranslated region was amplified using primers venushapX-f/hapX3′-r (PCR3, 1.6 kb; template: phapX^R^-hph). Subsequently, the *hapX* promoter region and Venus coding sequence were combined via fusion PCR using primers 5′hapX-f2/venushapX-r (PCR4, 2.1 kb; template: PCR1 & PCR2). In the final PCR, *hapX* promoter DNA linked to *venus* coding sequence was fused to *hapX* coding sequence including the 3′ terminator region with primers hapXtrunc-f/hapX-r (PCR5, 3.5 kb; template PCR3 & PCR4). The resulting DNA fragment was digested with *Xba*I/*Bst*BI and subcloned into *Xba*I/*Bst*BI opened phapX^R^-hph. The 5′*hapX* promoter region includes an *Xba*I recognition site and primer hapX-r contains the palindromic recognition sequence for *Bst*BI.

For transformation, 5 μg of the respective plasmid was linearized through digestion with SnaBI, the recognition site of which is located 761 bp downstream of the stop codon.

### Fluorescence microscopy, PpIX analysis, siderophore analysis, Northern analysis, and qRT-PCR analysis

For imaging of hyphae, cells were grown on coverslips in 0.3 ml AMM containing 1% (w/v) glucose, 20 mM glutamine and the desired iron concentration. Microscopy images were captured using an Axio Imager. M2 microscope (Carl Zeiss) equipped with a 63×  oil immersion objective lens (numerical aperture, 1.40), a HPX 120 V compact light source (Carl Zeiss) and the AxioCam MRm camera (Carl Zeiss). Images were processed using ZEN 2012 imaging software (Carl Zeiss). DAPI was used to stain nuclei. PpIX content, siderophore production, RNA isolation, and Northern analysis were carried out as described previously (Hortschansky *et al*, 2007). The hybridization probes used in this study were generated by PCR using DIG-labeled nucleotides. For qRT-PCR analysis, RNA was digested with DNase I and column eluted using RNA Clean & Concentrator™-25 kit (ZYMO Research). cDNA was synthesized using GoScript™ Reverse Transcription System (Promega) and random primers. qPCR was performed in a StepONE Plus Instrument (Applied Biosystems) with POWER SYBR® Green PCR Master Mix (Applied Biosystems). Primers for *hapX*, *sreA,* and *actA* are listed in Supplementary Table S4.

### Immunoprecipitation of Venus-HapX fusion protein from *A. fumigatus* cell extracts

For protein extraction from AfS77 (wt) and *hapX*^*VENUS*^ mycelia, a modification of a published procedure was used (Liu *et al*, 2010). Briefly, mycelia were washed, frozen in liquid nitrogen, lyophilized and homogenized by grinding with mortar and pestle. 100 mg cell powder was resuspended in 1 ml lysis buffer (25 mM Tris/HCl, 300 mM NaCl, 0.5% (v/v) NP-40, 5 mM EDTA, 15 mM EGTA, 1 mM AEBSF, 1 mM DTT, 1× cOmplete protease inhibitor (Roche), pH 7.5). Extracts were cleared by two centrifugation steps (30 and 10 min at 53,200 × *g* at 4°C). Twenty-five microliter of pre-equilibrated GFP-Trap agarose beads (ChromoTek) were added to the supernatant and incubated for 1 h on an end-over-end rotor at 4°C. The supernatant was removed by centrifugation, and the beads were washed four times with wash buffer (25 mM Tris/HCl, 300 mM NaCl, 5 mM EDTA, 1 mM AEBSF, 1× complete protease inhibitor (Roche), pH 7.5) followed by two washing steps with water. Bound proteins were eluted with 100 μl of 10% (v/v) acetonitrile, 5% (v/v) acetic acid. 50 μl of each protein eluate was dried down in a vacuum centrifuge, boiled in 1× SDS-PAGE sample buffer, and loaded onto 4–12% NuPAGE Bis-Tris gels. Proteins were transferred to a PVDF membrane using the iBlot system (Invitrogen). After antibody incubation, the membrane was developed using 1-Step Ultra TMB-Blotting substrate (Thermo). The following antibodies were used: anti-GFP antibody (ab290, Abcam); HRP-conjugated anti-rabbit IgG antibody (GGHL-15P, ICL, Inc.).

### Western blot detection of S-tagged HapX

For Western blots, 100 mg dry weight of lyophilized mycelia were rehydrated in 1 ml of TNETG buffer (20 mM Tris, pH 7.4, 2.5 mM EDTA, 150 mM NaCl, 10% (v/v) glycerol, 0.5% (v/v) Triton X-100, 1 mM PMSF). Cells were lysed by 5 bursts of 1 min each in the presence of 1/3 volume of glass beads. Cell debris were removed by centrifugation. For Western blots, 50 μg protein were loaded per lane and separated on a 12.5% SDS-PAGE. Blots were immuno-decorated with a polyclonal anti-S-tag antibody (ICL, Inc.) for detection of S-tagged HapX or a polyclonal antibody directed against *S. cerevisiae* Porin (Por1).

### MEME analysis

Protein blast (http://blast.ncbi.nlm.nih.gov/Blast.cgi) was used to identify the most similar homologs of *A. fumigatus* CccA (XP_751578.1). The 1-kb 5′-upstream sequences of the top 26 hits from unique species (Supplementary Fig S6) as well as *A. fumigatus* and *F. oxysporum,* which is known to require HapX for iron detoxification (see above), were subject to MEME analysis (Bailey & Elkan, 1994) to identify putative common motifs of size 6–35 bp, occurring once or zero times.

### Bacterial expression and purification of the CBC and HapX for SPR analysis

The *A. fumigatus* core CBC was produced and purified as described for the CBC from *A. nidulans* (Huber *et al*, 2012). Briefly, synthetic genes coding for HapC(40–137) and HapE(47-164) were cloned in the pnCS vector (Diebold *et al*, 2011) for expression of a bicistronic transcript. A synthetic gene encoding HapB(230–299) was cloned into pET39b (Novagen) and *E. coli* BL21(DE3) cells were co-transformed with both plasmids. After overnight autoinduction and cell lysis, the heterotrimeric CBC was purified to homogeneity by subsequent cobalt chelate affinity and size-exclusion chromatography.

A cDNA fragment encoding *A. fumigatus* HapX(24–158) (covering the CBC-binding domain, basic region, and coiled-coil domain) with an extended N-terminus including a cleavage site for tobacco etch virus (TEV) protease was amplified and subcloned into the pMAL-c2X (New England Biolabs) vector. The resulting plasmid was transformed into *E. coli* Rosetta 2 (DE3) cells for overnight autoinduction. Crude bacterial lysates were purified by Dextrin Sepharose affinity chromatography (GE Healthcare). The maltose-binding protein HapX(24–158) fusion was cleaved with TEV protease and further purified sequentially using CellufineSulfate (Millipore) affinity chromatography, (NH_4_)_2_SO_4_ precipitation (50% w/v), and Superdex 75 prep grade (GE Healthcare) size exclusion chromatography. The absolute molecular mass of HapX(24–158) was determined by static light scattering experiments on a miniDawn TREOS monitor in series with an Optilab T-rEX differential refractometer (Wyatt). HapX(24–158) was chromatographed on a Superdex 200 10/300 GL column (GE Healthcare), and molar mass was calculated using ASTRA 6 software (Wyatt).

### Surface plasmon resonance measurements

Real-time analyses were performed on a Biacore T200 system (GE Healthcare) at 25°C. DNA duplexes containing CCAAT box at position −369 in the 5′-upstream region of the *A. fumigatus cccA* gene were produced by annealing complementary oligonucleotides using a fivefold molar excess of the non-biotinylated oligonucleotide. The dsDNA was injected on flow cells of a streptavidin (Sigma)-coated CM3 sensor chip at a flow rate of 10 μl/min until the calculated amount of DNA that gives a maximum CBC-binding capacity of 100 RU had been bound. CBC and HapX(24–158) samples containing 10 μg/ml poly(dI-dC) were injected in running buffer (10 mM phosphate pH 7.4, containing 2.7 mM KCl, 137 mM NaCl, 1 mM DTT and 0.005% (v/v) surfactant P20) at a flow rate of 30 μl/min. Co-injection of HapX(24–158) on preformed binary CBC/DNA complexes within the equilibrium phase was performed by using the dual injection command. Each injection was performed at least two times. The chip surface was regenerated with 10 mM Tris/HCl pH 7.5, containing 0.5 M NaCl, 1 mM EDTA and 0.005% (w/v) SDS for 1 min. Refractive index errors due to bulk solvent effects were corrected with responses from DNA-free flow cell 1 as well as subtracting blank injections. Kinetic raw data were processed and globally fitted with Scrubber 2.0c (BioLogic Software) using a 1:1 interaction model including a mass transport term.

### ChIP analysis

1 × 10^6^ spores/ml of wild-type (AfS77) or the strain carrying Venus-tagged HapX (*hapX*^*VENUS*^) cultures were grown in liquid AMM media for 18 h at 37°C, then shifted to fresh media with no iron, 0.03 mM, or 3 mM iron for 8 h. ChIP was performed as previously described (Blatzer *et al*, 2011). Briefly, cells were exposed to 1% (v/v) formaldehyde for crosslinking, DNA was collected by lysis of powdered tissue in ChIP lysis buffer and sheared with sonication. ChIP was performed with 1 μg anti-GFP polyclonal antibody (ab290; Abcam) or IgG control on Dynabeads Protein A magnetic beads (Invitrogen). ChIP'd DNA was treated with RNase A and binding was assessed by qPCR. qPCR was performed in triplicate using 0.5 μl of ChIP'd DNA or input control DNA in a 20 μl reaction with 1× iQ Sybr Green Supermix and 0.2 mM final concentration of each primer. Cycle parameters for qPCR were 40 cycles of 94°C for 30 s and 60°C for 30 s, using BioRad iQ single color real-time PCR detection system. Percent input was calculated according to the Life Technologies ChIP analysis website. Briefly, for each sample, the input controls were adjusted to correct for amount of DNA template using [(mean input control Ct)-log_2_(100/30)]. Percent enrichment was calculated using 100*(2^(Adjusted inputCt-ChIPCt)^). As an additional negative control, a region of *actA* promoter region (nt −980 to −725 relative to the translation start) not predicted to bind HapX was PCR amplified. Oligonucleotides used for ChIP analysis are listed in Supplementary Table S4.
