# Supplementary material for: The Janus transcription factor HapX controls fungal adaptation to both iron starvation and iron excess
Source: EMBO J. 2014 Aug 4;33(19):2261–76. doi: 10.15252/embj.201489468 (PMC4232046; doi:10.15252/embj.201489468)
Supplement: Supplementary file 1 [file embj0033-2261-sd1.pdf]

THE
EMBO
JOURNAL

Manuscript EMBO-2014-89468

The Janus transcription factor HapX controls fungal adaptation to both iron starvation and iron excess

Fabio Gsaller, Peter Hortschansky, Sarah R. Beattie, Veronika Klammer, Katja Tuppatsch, Beatrix E. Lechner, Nicole Rietzschel, Ernst R. Werner, Aaron A. Vogan, Dawoon Chung, Ulrich Mühlenhoff, Masashi Kato, Robert A. Cramer, Axel A. Brakhage and Hubertus Haas

Corresponding author: Hubertus Haas, Innsbruck Medical University

Review timeline:

Submission date:	09 January 2014
Editorial Decision:	10 February 2014
Revision received:	09 May 2014
Editorial Decision:	22 May 2014
Appeal:	23 May 2014
Editorial Decision:	26 May 2014
Additional Author Correspondence:	26 May 2014
Additional Editorial Correspondence:	26 May 2014
Resubmission:	08 July 2014
Editorial Decision:	09 July 2014
Revision:	15 July 2014
Accepted:	15 July 2014

Transaction Report:

(Note: With the exception of the correction of typographical or spelling errors that could be a source of ambiguity, letters and reports are not edited. The original formatting of letters and referee reports may not be reflected in this compilation.)

Editor: David del Alamo

1st Editorial Decision 10 February 2014

Thank you for the submission of your manuscript entitled "The Janus transcription factor HapX controls fungal adaptation to both iron starvation & iron excess". We have now received the full set of reports from the referees that were asked to evaluate your study, which I copy below.

As you will see below, despite the rather negative opinion of referee #2, referees #1 and #3 consider that your manuscript is of high novelty and interest and we have therefore decided to invite you to submit a revised version of your manuscript to The EMBO Journal.

Without going into details that you will find below, and besides other technical points that you will need to address, both referee #1 and #3 concur in the absolute necessity of solving the question of HapX expression under physiological or high iron conditions. I would like to draw your attention to this issue, as it will be key for the acceptance of your manuscript. In any case, do not hesitate to

contact me by e-mail or on the phone if you have any questions, you need further input or you anticipate any problems during the revision process.

Please be aware that it is 'The EMBO Journal' policy to allow a single round of major revision only and that we generally allow three months as standard revision time. As a matter of policy, competing manuscripts published during this period will not be taken into consideration in our assessment of the novelty presented by your study ("scooping" protection). Nevertheless, please contact me as soon as possible upon publication of any related work in order to discuss how to proceed. Should you foresee a problem in meeting this three-month deadline, please let us know in advance and we may be able to grant an extension.

When preparing your letter of response to the referees' comments, bear in mind that this will form part of the Review Process File, and will therefore be available online to the community. For more details on our Transparent Editorial Process initiative, please visit our website: http://emboj.msubmit.net/html/emboj_author_instructions.html#a2.12

Thank you very much again for the opportunity to consider your work for publication. I look forward to your revision.

REFEREE REPORTS:

Referee #1:

This manuscript continues the characterization of the regulatory factor HapX from Aspergillus fumigatus. HapX is a regulatory subunit of the CCAAT-binding factor that is known to be expressed under low iron (Fe) conditions. Under Fe deficiency, HapX associates with the CCAAT-binding core complex and mediates repression of genes encoding iron-using proteins. In this study, the authors provide genetic evidence that HapX is also required in cells undergoing a transition from low to high iron in which it mediates the transcriptional activation of the vacuolar iron transporter CccA, therefore increasing resistance to excess iron.

Major points.

The data supporting a direct action by HapX would be greatly strengthened if the authors can clearly demonstrate the presence of HapX when cells are shifted from low Fe (-Fe) to high Fe (sFe). Under Fe-replete conditions (sFe, +Fe, or hFe), results show that neither hapX transcript (Figs 1A, 4D and 5C) nor HapX protein (Figs 3 and 4E (missing panel)) are present. How HapX could be directly involved in the proposed mechanism if the protein itself is absent (no detection of HapX what-so-ever) (Figs 3 and 4E (missing panel under Fe-replete conditions)).
Because of that, the authors should show the HapX-Venus fusion protein was indeed expressed and seen in ChIP assays under both low and high levels of iron (Fig. 7C). Cell lysates that contain HapX-Venus cross-linked to chromatin by formaldehyde should be analyzed by immunoblotting (as well as unbound HapX-Venus). Irrespective of whether the proteins are cross-linked to chromatin or not, HapX-Venus should be clearly produced under both iron-limiting and iron-replete conditions.

Additional points.

1. In Fig. 1, the authors should define the "sFe" treatment as they did in their previous publication in Metallomics (2012, 4:1262-70). It is somewhat surprising to observe very low levels of cccA expression in a hapX null under Fe-replete conditions (+Fe, 30 uM), any explanation for that? In Fig. 1, the authors should indicate that the Fe-sensitive phenotype of cccA null cells was analyzed in Fig. 2 and in Metallomics (2012, 4:1262-70). In Fig. 1, the authors should also indicate that hapX mRNA levels under "sFe" conditions were analyzed in Fig. 4. As a reader, we look for those results in Fig. 1.

2. In Fig. 3, the cellular localization of HapX-Venus is not shown after a shift from low (-Fe) to high Fe (+Fe), in other words under "sFe" treatment. Furthermore, on the copy of the manuscript, there is no evidence of nuclear localization of HapX-Venus. How this result could correlate with the ChIP obtained in Fig. 7C (HapX-Venus +Fe)?
In Fig. 3, HapX-Venus should be analyzed by Western blots.

In page 7, second paragraph, there is an incomplete sentence related to this figure (thus far, nuclear localization is known to be seen under low Fe conditions).

3. In Fig. 4E, Western blot analysis under Fe-replete conditions is missing.

4. Page 7 (bottom), does "CR" mean "Cys residue"?

5. In Fig. 5, one interpretation of the hapX158 phenotype is that the protein was not produced since the presence of the protein is unclear. No molecular marker is shown to help the reader to judge about the size of the polypeptides.
More importantly, what about a truncated construct which has only the CRR-A domain (e.g. 1 to 226 AAs), but not the CRR-B domain?
Page 9, the authors concluded that the presence of CRR-A and CRR-B are required for Fe resistance, however they have not tested those domains as single domain in their functional assays.

6. Results do not suggest an important functional role for CRR-C, why the authors attribute a functional importance to this domain (see page 8, end of second paragraph)?

7. Pages 8-9, one interpretation of the phenotype generated by the hapXC115A mutant is that the protein is not produced since in Fig. 4 (line 4) there is absence of the mutant protein. Therefore, absence of protein (HapXC115A) would be consistent with a phenotype produced by a HapX null strain. It would be critical to detect the protein before reaching any conclusion about the functional importance of Cys115.

8. One recommendation would be to move text starting at - "An evolutionary conserved cccA promoter element..." (pages 12 and 13) immediately before the paragraph entitled "Both functions, adaptation to iron limitation..." page 10.

Referee #2:

This is a technically sound study regarding the HapX transcription factor from Aspergillus fumigatus and its interesting dual role in both activating and repressing transcription in response to extremes in iron starvation versus iron excess. The data is well presented and the conclusions match the observations made. My only concern is whether this paper is suitable for the wide readership of EMBO J.

Referee #3:

In this manuscript the authors examine the function of the HapX transcription factor of Aspergillus spp. HapX has been characterized as a CCAAT-box binding TF involved in repressing transcription of genes involved in iron utilization during times of iron-limited growth. HapX also appears to be required to activate transcription of a siderophore transporter during iron deficiency and both repress transcription during iron deficiency and activate transcription during iron excess for an iron efflux pump. In a series of carefully controlled analyses, HapX is shown to function in the response to iron excess, despite no evidence of HapX protein being present in these conditions. Site-directed mutagenesis studies examined the roles of conserved clusters of cysteine residues in the C-terminus of HapX. Alanine substitution of cysteines in two clusters was associated with specific loss of the functions of HapX in iron-excess, without affecting HapX function in iron deficiency. Truncation of the C-terminus was shown to affect HapX functions in iron deficiency without affecting iron-excess function. The sequences recognized by HapX in the cccA promoter consisted of a canonical CCAAT box and also a second adjacent sequence conserved in cccA promoters across HapX-containing fungal species. Surprisingly, HapX occupancy at the cccA promoter was readily

detectable in both iron-deficient (when HapX expression is high) and iron-sufficient and iron-excess conditions (when HapX is not detectable).

The authors have presented some interesting and intriguing data regarding the complex activities of HapX. Their data suggest that multiple regulatory domains exist in HapX and they provide strong experimental evidence in the form of site-direct mutagenesis and truncation mutations expressed at the endogenous locus under the native promoter. My greatest concern, however, is the lack of evidence that HapX protein is expressed at all under the growth conditions described in the manuscript. HapX mRNA was not detected in the iron-sufficient or iron-excess conditions and the HapX protein was similarly not detected under these conditions. Although the site-directed mutagenesis data strongly suggest that HapX protein is mediating these effects, the reader is left to question whether a transcript from the HapX locus or an alternatively-spliced version of HapX could be mediating these effects. It is even more troubling to see that ChIP analysis suggests very similar amounts of HapX are bound to cccA promoter under all three iron conditions. Given that the authors have constructed N- and C-terminally tagged versions of HapX, they have the tools in hand to address this question. If the author's hypothesis is that very low levels of HapX protein are sufficient for its transcriptional effects in iron-replete conditions, perhaps isolation of nuclei or immunoprecipitation could be used to detect low levels of HapX. The authors do not present evidence to explain why some promoter interactions result in repression and some in activation.

Other concerns:

1. The 4th and 5th sentences of the abstract are confusing and poorly worded.

2. On p.8 descriptions of data in Fig. 4 include mutants not presented in the figure. Although the authors mention in the figure legend that these data are in the supplement, some mention of the data in the supplement needs to appear in the text.

3. The hapXC115A mutant did not accumulate in cells at all, likely because it failed to properly fold and was degraded. The authors cannot assert that this cysteine residue was important for grow in iron limitation, only that is was necessary for folding and could not be further evaluated.

4. There is evidence that S. cerevisiae Hap4 does participate in iron regulation. Please see J. Ihrig, A. Hausmann, A. Hain, N. Richter, I. Hamza, R. Lill, U. Muhlenhoff, Iron regulation through the back door: iron-dependent metabolite levels contribute
to transcriptional adaptation to iron deprivation in Saccharomyces cerevisiae,
Eukaryot. Cell 9 (2010) 460-471.

5. This manuscript merges the results and discussion sections and divides the structure/function analysis of HapX from the promoter analysis. To this reader, the separation seems artificial, and the paper might be better organized by moving the last three paragraphs of page 11 to the discussion section.

6. Given that the paper is about the specific activities of a transcription factor and that post-transcriptional regulation may also occur, the authors should be precise in using the terms "expression" and "transcription." For example, on p.13, last paragraph, the phrase "...cccA promoter suggests that expression of cccA is..." would be more precise if the term "transcription" was used instead of "expression".

1st Revision - authors' response 09 May 2014

Referee #1:

This manuscript continues the characterization of the regulatory factor HapX from Aspergillus fumigatus. HapX is a regulatory subunit of the CCAAT-binding factor that is known to be expressed

under low iron (Fe) conditions. Under Fe deficiency, HapX associates with the CCAAT-binding core complex and mediates repression of genes encoding iron-using proteins. In this study, the authors provide genetic evidence that HapX is also required in cells undergoing a transition from low to high iron in which it mediates the transcriptional activation of the vacuolar iron transporter CccA, therefore increasing resistance to excess iron.

Major points.

The data supporting a direct action by HapX would be greatly strengthened if the authors can clearly demonstrate the presence of HapX when cells are shifted from low Fe (-Fe) to high Fe (sFe). Under Fe-replete conditions (sFe, +Fe, or hFe), results show that neither hapX transcript (Figs 1A, 4D and 5C) nor HapX protein (Figs 3 and 4E (missing panel)) are present. How HapX could be directly involved in the proposed mechanism if the protein itself is absent (no detection of HapX what-so-ever) (Figs 3 and 4E (missing panel under Fe-replete conditions)).

Because of that, the authors should show the HapX-Venus fusion protein was indeed expressed and seen in ChIP assays under both low and high levels of iron (Fig. 7C). Cell lysates that contain HapX-Venus cross-linked to chromatin by formaldehyde should be analyzed by immunoblotting (as well as unbound HapX-Venus). Irrespective of whether the proteins are cross-linked to chromatin or not, HapX-Venus should be clearly produced under both iron-limiting and iron-replete conditions.

The original manuscript already contained several lines of evidence for the presence of HapX during high iron conditions: (i) the growth defect of the mutant lacking HapX not only during iron starvation but also during high iron conditions, (ii) the growth defect during high iron but not iron starvation conditions of mutants carrying cysteine-to-alanine mutations in two HapX cysteine rich regions (CRR-A and B), and (iii) the ChIP-qPCR-data demonstrating that HapX is bound independent of the environmental iron availability to an evolutionary conserved HapX/CBC binding motif in the promoter of the gene encoding CccA, which is the main target for HapX during high iron conditions to mediate iron resistance. In the original manuscript we were able to detect S-tagged HapX only under iron starvation but not physiological and high iron conditions. Using GFP-trap (an enrichment method for GFP-tagged proteins) combined with Western blot analysis, we were now able to detect VENUS-HapX fusion protein (produced under control of the enogenous *hapX* promoter) also under physiological and high iron conditions. The data are shown in the new Figure 3B and are discussed in the text. The S-tag protein detection method was obviously not sensitive enough to detect HapX.

Additional points.

1. In Fig. 1, the authors should define the "sFe" treatment as they did in their previous publication in Metallomics (2012, 4:1262-70).

Thank you for the hint, corrected as suggested!

It is somewhat surprising to observe very low levels of cccA expression in a hapX null under Fe-replete conditions (+Fe, 30 uM), any explanation for that?

The wild type lacks transcription of *cccA* under iron-replete conditions (+Fe, 30 uM). The low *cccA* transcript level of the mutant during this condition might indicate slight derepression (as found to a higher extent during -Fe), possibly due to the fact that cells lacking HapX are faster iron-starved compared to wild type because HapX is required to avoid cellular iron starvation by controlling iron uptake and iron consumption.

In Fig. 1, the authors should indicate that the Fe-sensitive phenotype of cccA null cells was analyzed in Fig. 2 and in Metallomics (2012, 4:1262-70).

Corrected as suggested!

In Fig. 1, the authors should also indicate that hapX mRNA levels under "sFe" conditions were analyzed in Fig. 4. As a reader, we look for those results in Fig. 1.

Corrected as suggested!

2. In Fig. 3, the cellular localization of HapX-Venus is not shown after a shift from low (-Fe) to high Fe (+Fe), in other words under "sFe" treatment. Furthermore, on the copy of the manuscript, there is no evidence of nuclear localization of HapX-Venus. How this result could correlate with the ChIP obtained in Fig. 7C (HapX-Venus +Fe)?

In Fig. 3, HapX-Venus should be analyzed by Western blots.

The epifluorescence microscopy analysis shown in Figure 3 was replaced by a new analysis, now clearly showing (i) nuclear localization of $HapX^{VENUS}$ (N-terminal Venus-tagged HapX) during iron starvation (Figure 3A), (ii) the response of Venus-HapX localization to an one-hour shift from iron starvation to sufficiency (sFe) (Figure 3A), and (iii) detection of $HapX^{VENUS}$ by Western blot after GFP-trap enrichment during iron starvation, iron sufficiency and high iron conditions (Figure 3B).

The results are discussed in the text.

In page 7, second paragraph, there is an incomplete sentence related to this figure (thus far, nuclear localization is known to be seen under low Fe conditions).

Corrected! Moreover, the results of Figure 3B are now discussed in the 2nd paragraph on page 7.

3. In Fig. 4E, Western blot analysis under Fe-replete conditions is missing.

This analysis was performed, however, we were unable to detect S-tagged HapX during +Fe and sFe conditions (even after immunoprecipitation of S-tagged proteins) and therefore the results were not shown. Obviously, the sensitivity of this method is not sufficient for the low HapX protein amount present under these conditions.

This was mentioned in the original manuscript in the 2nd paragraph on page 7. To increase clarity, we now mentioned this also in the legend to Figure 4E.

4. Page 7 (bottom), does "CR" mean "Cys residue"?

Yes, to improve readability we replaced ”CR” by ”Cys”.

5. In Fig. 5, one interpretation of the hapX158 phenotype is that the protein was not produced since the presence of the protein is unclear. No molecular marker is shown to help the reader to judge about the size of the polypeptides.

Figure 5C shows a Northern analysis and the reduction in transcript sizes roughly resembles the extent of C-terminal HapX truncation. Western blot analysis of the truncated HapX versions is not feasible as these proteins do not carry an S-tag. Of course, the phenotypes seen might be generally a mixture of the change in expression level and truncation. What makes it in particular difficult is that HapX is most likely autoregulated and *hapX* overexpression is toxic (data not shown). For the latter reason all constructs were placed as single copy at the original *hapX* locus. It is, however, highly unlikely that there is no protein in the $hapX^{158}$ mutant as there are clear differences in its phenotype compared to *DhapX* under iron starvation conditions: higher biomass production, higher TAFC production, higher *mirB* expression and lower PpIX accumulation. In contrast to the other truncations, $hapX^{158}$ shows largely the same iron sensitivity as *DhapX*, most likely due to the lack of CCR-A and -B regions, that are present in the other truncated versions.

More importantly, what about a truncated construct which has only the CRR-A domain (e.g. 1 to 226 AAs), but not the CRR-B domain?

Page 9, the authors concluded that the presence of CRR-A and CRR-B are required for Fe resistance, however they have not tested those domains as single domain in their functional assays.

The goal of the truncation experiments was the functional analysis of the HapX C-terminus and not of the CRR domains - CRR functions were analyzed by site directed mutagenesis of 11 different cysteine residues (Figure 4 and 7S).

6. Results do not suggest an important functional role for CRR-C, why the authors attribute a functional importance to this domain (see page 8, end of second paragraph)?

Mutations in CRR-C (strains $hapXC2^{C350A}$ and $hapXC3^{C353A}$) led to a slightly decreased growth on solid medium and in liquid high iron media, but did not affect the transcription pattern of *cccA* and *leuA* (Figure 4). The statement was now clarified.

7. Pages 8-9, one interpretation of the phenotype generated by the hapXC115A mutant is that the protein is not produced since in Fig. 4 (line 4) there is absence of the mutant protein. Therefore, absence of protein (HapXC115A) would be consistent with a phenotype produced by a HapX null strain. It would be critical to detect the protein before reaching any conclusion about the functional importance of Cys115. We reworded the description.

Indeed, the $hapX^{C115A}$ mutation results in the loss of the HapX protein, a reduced *hapX* transcript level and a phenotype that resembles that of *DhapX*. Therefore, the most likely explanation is that the mutation results in the loss of HapX protein. The results are nevertheless interesting to show because the decrease of the *hapX* transcript level in this mutant indicates transcriptional autoregulation of HapX. The description of this part was reworded.

8. One recommendation would be to move text starting at - "An evolutionary conserved cccA

promoter element..." (pages 12 and 13) immediately before the paragraph entitled "Both functions, adaptation to iron limitation..." page 10.

Changed as suggested. Consequently, former Figure 6 is know Figure 7, and vice versa.

Referee #2:

This is a technically sound study regarding the HapX transcription factor from Aspergillus fumigatus and its interesting dual role in both activating and repressing transcription in response to extremes in iron starvation versus iron excess. The data is well presented and the conclusions match the observations made. My only concern is whether this paper is suitable for the wide readership of EMBO J.

HapX is a central iron regulator conserved in most fungal species and shown to be crucial for virulence of animal and plant pathogenic fungi. Therefore, we believe that detailed characterization of iron regulation and sensing is of high and broad interest not only from a basic science view. Moreover, the activating and repressing functions that depend on ambient iron availability make HapX a particular interesting model for gene regulation. Its function under iron starvation has been studied in different species including *A. nidulans, A. fumigatus, C. neoformans, C. albicans,* and *F. oxysporum* but its important function during iron excess has been overlooked until now. To the best of our knowledge, until today, there is no study elucidating the regulation in response to iron excess in fungal species apard from *S. cerevisiae*.

Referee #3:

In this manuscript the authors examine the function of the HapX transcription factor of Aspergillus spp. HapX has been characterized as a CCAAT-box binding TF involved in repressing transcription of genes involved in iron utilization during times of iron-limited growth. HapX also appears to be required to activate transcription of a siderophore transporter during iron deficiency and both repress transcription during iron deficiency and activate transcription during iron excess for an iron efflux pump. In a series of carefully controlled analyses, HapX is shown to function in the response to iron excess, despite no evidence of HapX protein being present in these conditions. Site-directed mutagenesis studies examined the roles of conserved clusters of cysteine residues in the C-terminus of HapX. Alanine substitution of cysteines in two clusters was associated with specific loss of the functions of HapX in iron-excess, without affecting HapX function in iron deficiency. Truncation of the C-terminus was shown to affect HapX functions in iron deficiency without affecting iron-excess function. The sequences recognized by HapX in the cccA promoter consisted of a canonical CCAAT box and also a second adjacent sequence conserved in cccA promoters across HapX-containing fungal species. Surprisingly, HapX occupancy at the cccA promoter was readily detectable in both iron-deficient (when HapX expression is high) and iron-sufficient and iron-excess conditions (when HapX is not detectable).

The authors have presented some interesting and intriguing data regarding the complex activities of HapX. Their data suggest that multiple regulatory domains exist in HapX and they provide strong experimental evidence in the form of site-direct mutagenesis and truncation mutations expressed at the endogenous locus under the native promoter. My greatest concern, however, is the lack of evidence that HapX protein is expressed at all under the growth conditions described in the

manuscript. HapX mRNA was not detected in the iron-sufficient or iron-excess conditions and the HapX protein was similarly not detected under these conditions. Although the site-directed mutagenesis data strongly suggest that HapX protein is mediating these effects, the reader is left to question whether a transcript from the HapX locus or an alternatively-spliced version of HapX could be mediating these effects. It is even more troubling to see that ChIP analysis suggests very similar amounts of HapX are bound to cccA promoter under all three iron conditions. Given that the authors have constructed N- and C-terminally tagged versions of HapX, they have the tools in hand to address this question. If the author's hypothesis is that very low levels of HapX protein are sufficient for its transcriptional effects in iron-replete conditions, perhaps isolation of nuclei or immunoprecipitation could be used to detect low levels of HapX.

The original manuscript already contained several lines of evidence for the presence of HapX during high iron conditions: (i) the growth defect of the mutant lacking HapX not only during iron starvation but also during high iron conditions, (ii) the growth defect during high iron but not iron starvation conditions of mutants carrying cysteine-to-alanine mutations in two HapX cysteine rich regions (CRR-A and B) and (iii) the ChIP-qPCR-data demonstrating that HapX is bound independent of the environmental iron availability to an evolutionary conserved HapX/CBC binding motif in the promoter of the gene encoding CccA, which is the main target for HapX during high iron conditions to mediate iron resistance. In the original manuscript we were able to detect S-tagged HapX only under iron starvation but not under physiological and high iron conditions. Using GFP-trap (an enrichment method for GFP-tagged proteins) combined with Western blot analysis, we were now able to detect VENUS-HapX fusion protein (produced under control of the endogenous *hapX* promoter) also under physiological and high iron conditions. The data are shown in the new Figure 3B and are discussed in the text (page 7/2nd paragraph). The S-tag protein detection method was obviously not sensitive enough to detect HapX.

The authors do not present evidence to explain why some promoter interactions result in repression and some in activation.

This is indeed an intriguing question. At the moment, we can only speculate on this. A discussion is found on page 14/2nd paragraph. Here, we also discuss recent findings of the link between sulfur and iron homeostasis (Amich et al, 2013; *PLoS Pathog* **9:** e1003573).

Other concerns:

1. The 4th and 5th sentences of the abstract are confusing and poorly worded.

These sentences were reworded:

"We identified HapX protein domains that are essential for HapX functions during either iron starvation or high iron conditions. The evolutionary conservation of these domains indicates a role in iron sensing. We further demonstrate that a HapX homodimer and the CCAAT-binding complex (CBC) cooperatively bind an evolutionary conserved DNA motif in a target promoter."

2. On p.8 descriptions of data in Fig. 4 include mutants not presented in the figure. Although the authors mention in the figure legend that these data are in the supplement, some mention of the data in the supplement needs to appear in the text.

We added the sentence ”For simplicity, in Figure 4 only one mutant per CRR is shown, the respective, phenotypically identical second mutant is shown in Figure S7.” (page 8, 2nd paragraph). Moreover, we referenced to Figure S7 where appropriate.

3. The hapXC115A mutant did not accumulate in cells at all, likely because it failed to properly fold and was degraded. The authors cannot assert that this cysteine residue was important for grow in iron limitation, only that is was necessary for folding and could not be further evaluated.

The respective text was reworded and the title of Figure 4 was changed.

4. There is evidence that S. cerevisiae Hap4 does participate in iron regulation. Please see J. Ihrig, A. Hausmann, A. Hain, N. Richter, I. Hamza, R. Lill, U. Muhlenhoff, Iron regulation through the back door: iron-dependent metabolite levels contribute to transcriptional adaptation to iron deprivation in Saccharomyces cerevisiae, Eukaryot. Cell 9 (2010) 460-471.

A reference to this study was included and further discussed.

5. This manuscript merges the results and discussion sections and divides the structure/function analysis of HapX from the promoter analysis. To this reader, the separation seems artificial, and the paper might be better organized by moving the last three paragraphs of page 11 to the discussion section.

As suggested by Referee #1, the section **”An evolutionary conserved cccA promoter element..."** (pages 12 and 13) was moved directly in front of the paragraph entitled **"Both functions, adaptation to iron limitation...".** Consequently, former Figure 6 is know Figure 7, and vice versa.

6. Given that the paper is about the specific activities of a transcription factor and that post-transcriptional regulation may also occur, the authors should be precise in using the terms "expression" and "transcription." For example, on p.13, last paragraph, the phrase "...cccA promoter suggests that expression of cccA is..." would be more precise if the term "transcription" was used instead of "expression".

Thanks a lot for the hint, corrected.

2nd Editorial Decision 22 May 2014

Thank you for the submission of your revised manuscript to The EMBO Journal. We have now received the comments from former referee #1 (copied below), and I am sorry to say that his/her assessment is not supportive of publication.

As the referee report is rather straightforward, I will not repeat his/her detailed arguments here. In brief, although s/he acknowledges that the manuscript has seen some improvements compared to the previous version, there is one fundamental concern already raised in the first round of review (also by referee #3) and that has not been properly addressed: it remains unclear whether HapX is actually expressed under physiological or high iron conditions. As I already mentioned in my decision letter, and based on the comments from the referees, this issue is key for the acceptance of your

manuscript.

Given the negative nature of the evaluation of you manuscript by the referee, that a major issue previously raised still remains, and the fact that it is The EMBO Journal policy to allow for a single major revision, I am afraid that we cannot call for yet another revised version of your manuscript at this stage and therefore we cannot offer to publish it.

That being said, however, if you feel that you can obtain data that would definite and conclusively address the issue of HapX expression, then we would have no objection to consider a new manuscript in the near future. To be completely clear, however, I have to inform you that a new manuscript would need to be treated as a new submission rather than a revision and, while we will try to contact the same referees, it would be reviewed afresh, also with respect to the literature and the novelty of your findings at the time of resubmission.

I am sorry that I have to disappoint you at this later stage. I hope, however, that the referee comments will be helpful and I thank you once more for the opportunity to consider your manuscript.

REFEREE REPORT:

Referee #1:

This is the revised version of the manuscript entitled "The Janus transcription factor HapX controls fungal adaptation to both iron starvation and iron excess".

Major points were as follows:

The data supporting a direct action by HapX would be greatly strengthened if the authors can clearly demonstrate the presence of HapX when cells are shifted from low Fe (-Fe) to high Fe (sFe). Under Fe-replete conditions (sFe, +Fe, or hFe), results show that neither hapX transcript (Figs 1A, 4D and 5C) nor HapX protein (Figs 3 and 4E (missing panel)) are present. How HapX could be directly involved in the proposed mechanism if the protein itself is absent (no detection of HapX what-so-ever) (Figs 3 and 4E (missing panel under Fe-replete conditions)).

Except for the addition of Fig. 3B, none of them have been corrected or modified. Then, the authors should not claim what they are not able to show. Therefore, the manuscript should be modified, otherwise everything is overstated.

Because of that, the authors should show the HapX-Venus fusion protein was indeed expressed and seen in ChIP assays under both low and high levels of iron (Fig. 7C). Cell lysates that contain HapX-Venus cross-linked to chromatin by formaldehyde should be analyzed by immunoblotting (as well as unbound HapX-Venus). Irrespective of whether the proteins are cross-linked to chromatin or not, HapX-Venus should be clearly produced under both iron-limiting and iron-replete conditions.

Again, no additional data has been added. As mentioned by the reviewer 3, surprisingly, HapX occupancy at the cccA promoter was readily detectable under iron-deficient, iron-sufficient and iron-excess conditions. However, the authors do not show the presence of the HapX protein as requested by both reviewers.

In Fig. 3, there is no evidence of nuclear localization of HapX-Venus. How this result could correlate with the new panel 3B and the ChIP results obtained in Fig. 7C (HapX-Venus +Fe, hFe, and sFe)?

In Fig. 5, one interpretation of the hapX158 phenotype is that the protein was not produced since the presence of the protein is unclear.

Only speculation has been suggested in the rebuttal letter and no correction has been incorporated in the revised version of the manuscript. Again, this point is clearly overstated.

More importantly, what about a truncated construct which has only the CRR-A domain (e.g. 1 to 226 AAs), but not the CRR-B domain?
Page 9, the authors concluded that the presence of CRR-A and CRR-B are required for Fe resistance, however they have not tested those domains as single domain in their functional assays.

This point is still unanswered. Fig. 7S is far away to be informative as compared with Fig 5, which itself, is still inconclusive versus the overstatement of the authors.

Appeal 23 May 2014

We are surprised and disturbed about the negative decision - I hope it is based on a misunderstanding, the wording of our data description or a potential confusion of the original submission and the revised version!

The fundamental concern raised in the first round of review by both referees was the lack of demonstration for the presence of Venus-HapX fusion protein under physiological and high iron availability (from your response letter: "....both referee #1 and #3 concur in the absolute necessity of solving the question of HapX expression under physiological or high iron conditions. I would like to draw your attention to this issue, as it will be key for the acceptance of your manuscript"). Of course we addressed this issue experimentally. In the revised version we now present clear proof for the presence of Venus-HapX fusion protein under physiological and high iron availability in Aspergillus fumigatus cell extracts by Western blot analysis (shown in the new Figure 3B - please see attached file). This is in perfect agreement with the permanent, iron-independent occupancy of the promoter of the HapX target gene cccA (ChIP) data) and the phenotypes of hapX mutants that lack the entire gene or cysteine residues found to be essential for growth during high iron conditions.

The data presented in Fig. 3B are described and discussed in the second paragraph at page 7 (please see highlighted passage at page 7 in the attached revised manuscript)! We outlined these data in the cover letter as well as in the point-by-point response. It is not clear for us, why referee #1 and you do not acknowledge and even ignore these new data?

Please do not hesitate to contact me by e-mail or on the phone if you have any questions!
Sincerely,

3rd Editorial Decision 26 May 2014

I have reviewed again referee #1's comments and your reponses and I have discussed your case with other members of the editorial team and I have to partially agree with you. I think the confusion stems from the fact that the closer you get to detecting HapX under normal or high iron conditions is the detection of Venus-HapX, which even if it is under the control of the HapX promoter, it is not HapX itself.

As you correctly point out in your letter, I originally stated that detection of HapX under normal or high iron conditions is a requisite for the publication of your paper. And we still believe that at least the detection of the endogenous mRNA should be provided before your manuscript can be further considered. It is a question of physiological significance and HapX-Venus is not a physiological protein.

As mentioned before, I would be glad to accept your manuscript if you are able to detect the expression of the endogenous gene (at least the mRNA, see above) under normal or high iron conditions.

I am sorry to have to disappoint you again and I hope that this letter explains the rationale behind our decision.

Additional Author Correspondence 26 May 2014

Thank you for your fast response! I agree, the Venus-HapX protein is not identical with HapX. However,

(i) the strain expressing Venus-HapX instead of HapX behaves as the wild type during both iron starvation and iron excess (demonstrated in liquid and plate culture growth assays, target gene transcript profiling, target metabolite profiling: siderophores, protoporphyrin IX), i.e. it cures all defects caused by HapX-deficiency, which demonstrates the functionality of the fusion protein: so to my mind it is a physiological protein;

(ii) to tag proteins for detection is a generally accepted practice as long as the tagged versions show wild type functionality (see above): this approach was not questioned by the referees, actually it was asked for;

(iii) to my mind, the protein detection (including Venus-HapX) is way more convincing than the mRNA-detection.

If you are still not convinced that our Venus-HapX protein detection under physiological and high iron conditions is sufficient proof, we will set up a qRT-PCR based detection of hapX mRNA.

I assume that this will be considered within the revision process and will not be treated as a new submission?!

Additional Editorial Correspondence 26 May 2014

Thank you very much for your quick e-mail.

As I said before, we partially agree with you. But in our opinion, which is also in agreement with the referees, you are presenting a hypothesis that is somewhat contradictory with what has been previously published. And one of the reasons by which you go against what has been previously published is that no other group has been able to detect HapX under normal or high iron conditions. So, while we agree with you that usually a fusion protein under endogenous control would be enough, in this case we believe that the evidence presented should go further, as no one has been able to observe this before.

We understand that protein detection might be tricky as multiple factors (and mainly antibody affinity) might interfere particularly in conditions in which the protein is not very concentrated. However, PCR-mediated mRNA detection is in principle a more sensitive technique that could at least demonstrate that the endogenous HapX gene is being activated under normal or high iron concentration. This, together with the rest of evidence presented, makes for a very strong case, which it is only in your best interest.

Naturally, there is no need for an extra round of review and your manuscript will be considered a revision rather than a resubmission, although this is more a technicality than a practical concern.

Thank you again for your patience and I hope you share our view on the interest in demonstrating endogenous activation of HapX

Resubmission 08 July 2014

Please find enclosed the revised version of our manuscript entitled: "The Janus transcription factor HapX controls fungal adaptation to both iron starvation and iron excess (EMBOJ-2014-87869R1-Q)", which we would like to be considered for publication in ***EMBO* Journal** as a Research Article.

As discussed, we now included qRT-PCR quantification of *hapX* (and in comparison of *sreA*) during iron starvation, iron sufficiency, iron excess and a one-hour shift from iron starvation to iron sufficiency. The data confirm the iron regulation of *hapX* (and *sreA*) at the transcriptional level as seen in the previously included Northern analysis. Moreover, the increased sensitivity of this method now also clearly demonstrates *hapX* transcription during iron sufficiency and iron excess as expected from the genetic data (phenotyping of loss-of function mutant as well as site-directed mutagenized strains) and the GFP-trap/Western blot analysis of the VENUS-HapX fusion protein (the production of which is controlled by the endogenous *hapX* promoter at the *hapX* locus). The data are shown in the new Figure 3A and are discussed in the first paragraph of page seven (marked in grey). For better readability, we shifted two consecutive sentences from *"Results and Discussion* (page 13, 2nd paragraph, final two sentences)*"* to *"Conclusions"* (also marked in grey).

Our study uncovers and characterizes a novel regulatory mechanism mediating both iron resistance and adaptation to iron starvation by the same transcription factor complex with activating and repressing functions depending on ambient iron availability. Therefore, we expect this study to be of interest to a wide range of researchers working on iron homeostasis/regulation/sensing in particular and gene regulation in general.

We hope that our manuscript now meets the criteria for publication in *EMBO Journal*.

Thank you very much for your efforts!

4th Editorial Decision 09 July 2014

Thank you for the submission of your revised manuscript to The EMBO Journal and thank you for your patience during this somewhat extended editorial process. As discussed in previous communications, I believe that the new additions to the manuscript address all major concerns posed by the referees and your study is therefore ready for publication, provided that a few minor details are dealt with, as discussed below.

Browsing through the manuscript, I have also noticed a few small issues with data presentation. Micrographs throughout the manuscript (Figs. 1, 2, 4, 5, 7 and S7) lack scale bars, which we require for clarity. Furthermore, the statistical analysis of the results requires a more detailed description in some cases. As a guide, statistical analyses must be described either in the Materials and Methods section or in the legend of the figure to which they apply and will include a definition of the error bars used (see error bars in 1C), and the number of independent experiments performed. The statistical significance analysis tool used, if any, must be also clearly stated.

In addition, although we allow supplemental Materials and Methods, as in this case this section is rather reduced in size compared to the corresponding section in the main text and even includes references, it would be probably clearer if it is also included in the main text. Obviously, supplemental tables and figures can remain in the supplemental information as long as they are referred to from the main text.

Once these minor issues have been solved, I will be glad to accept your manuscript for publication in The EMBO Journal.

Every paper now includes a 'Synopsis', displayed on the html and freely accessible to all readers. The synopsis includes an image, that you have already provided, as well as 2-5 one-short-sentence

bullet points that summarize the article. I would appreciate if you could provide these bullet points.

I would also like to mention that we now encourage the publication of source data, particularly for electrophoretic gels and blots but also for numerical data in graphs, with the aim of making primary data more accessible and transparent to the reader. Although optional at the moment, would you be willing to provide a PDF file per figure that contains the original, uncropped and unprocessed scans of all or key gels used in the figures? The PDF files should be labeled with the appropriate figure/panel number, and should have molecular weight markers; further annotation could be useful but is not essential. Raw numerical data for graphs can be provided as Excel (or related) tables. The files will be published online with the article as supplementary "Source Data" files.

If you have any questions regarding this initiative or any other part of the publication process, please let me know.

Thank you very much again for your patience. I am looking forward to seeing the final version of your manuscript.
