# Supplementary material for: The Janus transcription factor HapX controls fungal adaptation to both iron starvation and iron excess
Source: EMBO J. 2014 Aug 4;33(19):2261–76. doi: 10.15252/embj.201489468 (PMC4232046; doi:10.15252/embj.201489468)
Supplement: Supplementary file 2 [file embj0033-2261-sd2.pdf]

Supplementary Information

The Janus transcription factor HapX controls fungal adaptation to both iron starvation and iron excess

Fabio Gsaller[1]°, Peter Hortschansky[2]°, Sarah R. Beattie[3], Veronika Klammer[1], Katja Tuppatsch[2], Beatrix E. Lechner[1], Nicole Rietzschel[4], Ernst R. Werner[5], Aaron A. Vogan[6], Dawoon Chung[3], Ulrich Mühlenhoff[4], Masashi Kato[7], Robert A. Cramer[3], Axel A. Brakhage[2]* and Hubertus Haas[1]*

[1]Division of Molecular Biology, Biocenter, Innsbruck Medical University, Austria; [2]Department of Molecular and Applied Microbiology, Leibniz Institute for Natural Product Research and Infection Biology (HKI), and Friedrich Schiller University Jena, Germany; [3]Department of Microbiology and Immunology, Geisel School of Medicine at Dartmouth Hanover, NH, USA; [4]Institut für Zytobiologie und Zytopathologie, Philipps-Universität Marburg, Germany; [5]Division of Biological Chemistry, Biocenter, Innsbruck Medical University, Austria; [6]Department of Biology, McMaster University, Hamilton, Canada; [7]Faculty of Agriculture, Meijo University, Nagoya, Japan.

* To whom correspondence should be addressed.

E-mail:	hubertus.haas@i-med.ac.at	axel.brakhage@hki-jena.de
Tel.:	+43/(0)512/9003-70205	Tel.: +49/(0)3641-5321001
Fax:	+43/(0)512-9003-73100	Fax: +49/(0)3641-5320802

° Authors contributed equally

Key words: iron, regulation, sensing, transcription factor complex, fungi, siderophores

Table S1. Biomass production, TAFC production and PpIX accumulation of mutant strains. Liquid cultures were grown under conditions of iron starvation (-Fe), iron sufficiency (+Fe, 0.03 mM $FeSO_4$) and high iron availability (hFe, 3 mM $FeSO_4$) at 37°C for 24 h. The data represent the mean ± SD from biological triplicates. Statistically significant differences compared to the wild type and *hapX*^{R}, respectively, are shown in red (two-tailed, unpaired T-test; $p<0.05$).

(A) Biomass production of Δ*cccA* and Δ*hapX*. Compared strains are *A. fumigatus* ATCC 46645 derivatives.

Strain	Biomass production (g)		
	-Fe	+Fe	hFe
wt	0.28±0.015	0.65±0.021	0.64±0.030
Δ*cccA*	0.28±0.016	0.64±0.021	0.42±0.026
Δ*hapX*	0.13±0.010	0.62±0.028	0.24±0.019

(B) Biomass production, TAFC production and PpIX content of Δ*hapX*, Δ*hapX*Δ*cccA* and *hapX*^{VENUS}. Compared strains are *A. fumigatus* AfS77 derivatives.

Strain	Biomass production (g)			TAFC production (Abs_{435nm}/biomass)	PpIX accumulation (pmol/mg protein)
	-Fe	+Fe	hFe	-Fe	-Fe
wt	0.19±0.014	0.59±0.019	0.59±0.026	0.44±0.028	2031.3±223.39
Δ*hapX*	0.11±0.013	0.57±0.030	0.19±0.020	0.19±0.016	11278.5±1323.96
Δ*hapX*Δ*cccA*	0.12±0.010	0.56±0.019	0.18±0.014	0.16±0.020	10966.1±394.46

Strain	Biomass production (g)		
	-Fe	+Fe	hFe
wt	0.18±0.025	0.63±0.042	0.45±0.031
Δ*hapX*	0.11±0.012	0.62±0.027	0.29±0.018
hapX^{VENUS}	0.19±0.024	0.61±0.015	0.47±0.034

(C) Biomass and TAFC production of strains harboring cysteine-mutations within the HapX protein. Compared strains are *A. fumigatus* AfS77 derivatives.

Strain	Biomass production (g)			TAFC production (Abs_{435nm}/biomass)
	-Fe	+Fe	hFe	-Fe
Δ*hapX*	0.09±0.014	0.60±0.031	0.21±0.015	0.13±0.008
hapX^R	0.20±0.019	0.61±0.040	0.61±0.042	0.50±0.036
hapX^{C115A}	0.09±0.007	0.58±0.007	0.21±0.010	0.10±0.018
hapX^{C126A}	0.17±0.019	0.56±0.020	0.61±0.029	0.43±0.040
hapXA2^{C203A}	0.20±0.002	0.58±0.030	0.36±0.016	0.54±0.034
hapXB3^{C286A}	0.20±0.016	0.59±0.036	0.29±0.013	0.50±0.026
hapXC3^{C353A}	0.21±0.014	0.54±0.024	0.57±0.011	0.54±0.036
hapXD2^{C380A}	0.20±0.018	0.57±0.028	0.63±0.024	0.55±0.015
hapX^{C422A}	0.19±0.013	0.52±0.032	0.62±0.013	0.54±0.045

(D) Biomass production, TAFC production and PpIX content of strains expressing C-terminal truncated HapX protein. Compared strains are *A. fumigatus* AfS77 derivatives.

Strain	Biomass production (g)			TAFC production (Abs_{435nm}/biomass)	PpIX accumulation (pmol/mg protein)
	-Fe	+Fe	hFe	-Fe	-Fe
wt	0.23±0.012	0.65±0.022	0.63±0.011	0.46±0.022	1122.3±120.56
hapX^{464}	0.13±0.005	0.65±0.037	0.65±0.011	0.16±0.018	8594.0±669.35
hapX^{398}	0.12±0.007	0.62±0.029	0.61±0.010	0.11±0.022	7763.1±424.41
hapX^{374}	0.11±0.001	0.66±0.032	0.64±0.013	0.09±0.016	7055.5±267.92
hapX^{296}	0.10±0.012	0.63±0.026	0.65±0.021	0.07±0.001	6761.4±228.61
hapX^{158}	0.15±0.009	0.59±0.031	0.18±0.002	0.14±0.005	5065.6±632.79
Δ*hapX*	0.11±0.006	0.62±0.027	0.20±0.005	0.08±0.006	9831.9±735.56

Table S2. Proteases that are transcriptionally repressed by iron (Schrettl et al, 2008; Schrettl et al, 2010).

Gene	Protease
AFUA_2G01250	serine peptidase, family S28, putative
AFUA_3G05450	glutamate carboxypeptidase, putative
AFUA_3G11400	aspartic endopeptidase Pep2
AFUA_5G03990	aspartyl aminopeptidase
AFUA_5G04330	aminopeptidase, putative
AFUA_5G07330	carboxypeptidase S1, putative
AFUA_5G09210	alkaline serine protease Alp2
AFUA_6G13540	carboxypeptidase Y (CpyA), putative
AFUA_5G13300	aspartic endopeptidase Pep1
AFUA_8G00410	methionine aminopeptidase, type II, putative
AFUA_8G04730	oligopeptidase family protein

Table S3. Strains used in this study.

Strain	Genotype	Reference
wt, ATCC 46645, clinical isolate		American Type Culture Collection
Δ*hapX*	ATCC 46645, Δ*hapX::ptrA*	(Schrettl et al, 2010)
Δ*hapXcccA*^{oe}	ATCC 46645, Δ*hapX::ptrA, cccA*^{oe}*, hph*	This study
Δ*cccA*	ATCC 46645, Δ*cccA::ptrA*	(Gsaller et al, 2012)
Δ*cccAcccA*^{oe}	ATCC 46645, Δ*cccA::ptrA, cccA*^{oe}*, hph*	(Gsaller et al, 2012)
Δ*sreA*	ATCC 46645, Δ*sreA::hph*	(Schrettl et al, 2008)
wt, AfS77	ATCC 46645, Δ*akuA::loxP*	(Hartmann et al, 2010)
Δ*hapX*	AfS77, Δ*hapX::ptrA*	This study
Δ*hapX*Δ*cccA*	AfS77, Δ*hapX::ptrA,* Δ*cccA::hph*	This study
**Amino acid substitution**		
hapX^{R}	AfS77, Δ*hapX::ptrA, hapX*^{Stag}*, hph*	This study
hapX^{C115A}	AfS77, Δ*hapX::ptrA, hapX*^{C115A}*, hph*	This study
hapX^{C126A}	AfS77, Δ*hapX::ptrA, hapX*^{C126A}*, hph*	This study
hapXA2^{C203A}	AfS77, Δ*hapX::ptrA, hapX*^{C203A}*, hph*	This study
hapXA2^{C208A}	AfS77, Δ*hapX::ptrA, hapX*^{C208A}*, hph*	This study
hapXB1^{C277A}	AfS77, Δ*hapX::ptrA, hapX*^{C277A}*, hph*	This study
hapXB3^{C286A}	AfS77, Δ*hapX::ptrA, hapX*^{C286A}*, hph*	This study
hapXC2^{C350A}	AfS77, Δ*hapX::ptrA, hapX*^{C350A}*, hph*	This study
hapXC3^{C353A}	AfS77, Δ*hapX::ptrA, hapX*^{C353A}*, hph*	This study
hapXD2^{C380A}	AfS77, Δ*hapX::ptrA, hapX*^{C380A}*, hph*	This study
hapXD3^{C389A}	AfS77, Δ*hapX::ptrA, hapX*^{C389A}*, hph*	This study
hapX^{C422A}	AfS77, Δ*hapX::ptrA, hapX*^{C422A}*, hph*	This study
**Truncated HapX**		
hapX^{464}	AfS77, Δ*hapX::ptrA, hapX*^{464}*, hph*	This study
hapX^{398}	AfS77, Δ*hapX::ptrA, hapX*^{398}*, hph*	This study
hapX^{374}	AfS77, Δ*hapX::ptrA, hapX*^{374}*, hph*	This study
hapX^{296}	AfS77, Δ*hapX::ptrA, hapX*^{296}*, hph*	This study
hapX^{158}	AfS77, Δ*hapX::ptrA, hapX*^{158}*, hph*	This study
**Venus tagged HapX**		
hapX^{VENUS}	AfS77, Δ*hapX::ptrA, hapX*^{VENUS}*, hph*	
***A. nidulans* strains**		
wt, BPU1	*pyrG89*;*biA*;*wA3*;*argB2*;*pyroA4*; pAR5 (*argB*); ArgB+	(Tanaka et al, 2002)
Δ*hapX*, BPUΔX1	*pyrG89*; *biA*; *wA3*; *argB2*; *pyroA4*; *hapX*Δ*::argB;* ArgB+	(Tanaka et al, 2002)
***F. oxysporum* strains**		
wt, *Fusarium oxysporum* f sp *lycopersici* race 2 - 4287		FGSC 9935
Δ*hapX, Fusarium oxysporum* f sp *lycopersici* race 2 - 4287	Δ*hapX::hph*	(Lopez-Berges et al, 2012)

Table S4. Oligonucleotides (5′→3′) used in this study.

(A) Disruption of *hapX* and *cccA* coding sequence.

***hapX* disruption**	
oAfhapX-1	AGCGACTATAGCCGGATG
oAfhapX-2	CCTTGGGTCTTGAAGCTTGCG
oAfhapX-3	TCATCTAGACTGCCCAAGCTTCATACC
oAfhapX-4	ATCAGAGCTGGAGAGGCA
oAfhapX-5	TGGAGTTCCGATTGGTGC
oAfhapX-6	ATCCCGCTTCTTTCACCC
oAoPtrA1	GAGGACCTGGACAAGTAC
oAoPtrA2	CATCGTGACCAGTGGTAC

***cccA* disruption**	
oAfcccA1	CAACTCGGGCTCTGGGTC
oAfcccA5	CGGTGTCCTTGGTATTGC
oAfcccAr4	TGGGGCCTGGTTAGCGTC
oAfcccA3	TTGTAAGCTTGATCGACCATGGTTC
oAfcccAr6	AAGGATGCCGCCAATGTG
oAfcccAr2	GGGAGATCTCGCTGGCAC
ohph14	GAAGATGTTGGCGACCTC
ohph15	GAGAGCCTGACCTATTGC

(B) Generation of phapX^{R}-hph.

oAfhapX-S1	CGATACTTTGACCGACGGTTTGCCGACAAAGGAGCTGGTGCAGGCGCTG
oAfhapX-S2	CGCCAGCACATGGACAGCTAATTTATCGCATCTCTGCTTGTAGCATCACTC
oAfhapX-1	AGCGACTATAGCCGGATG
oAfhapX-S3	CGACGGTTTGCCGACAAA
oAfhapX-S4	TTTATCGCATCTCTGCTTGTAG
oAfhapX-7	CGCAAGCTTCAAGACCCAAGG
oAfhapX-8	GGGTGAAAGAAGCGGGAT
ohyg-1	CCATTAACCTAGGTACAG
ohyg-2	CTCCACTCGACCTGCAGG

(C) Site directed mutagenesis.

oAfHapX-C115A.f	TCGCGAGGTGGAGCAGGCGCGCTCCGAGATGACCTGGTG
oAfHapX-C115A.r	ACCAGGTCATCTCGGAGCGCGCCTGCTCCACCTCGCGAG
oAfHapX-C126A.f	ACCTGGTGGCGCGATCGTGCGCATGCGTTGGAGAAGGAG
oAfHapX-C126A.r	TCCTTCTCCAACGCATGCGCACGATCGCGCCACCAGGTC
oAfHapX-C203A.f	GTTAGGCTGCAATGATGCATCCACATCGCATTGCC
oAfHapX-C203A.r	GGCAATGCGATGTGGATGCATCATTGCAGCCTAAC
oAfHapX-C208A.f	ATTGCTCCACATCGCATGCGCAGTGCATTGAGGATGC
oAfHapX-C208A.r	GCATCCTCAATGCACTGCGCATGCGATGTGGAGCAAT
oAfHapX-C277A.f	CCTGCCGTGGATCCCGCCGGCTTCTGTTCGGATGGC
oAfHapX-C277A.r	GCCATCCGAACAGAAGCCGGCGGGATCCACGGCAGG
oAfHapX-C286A.f	TTCGGATGGCACGCCGGCCGTTTGCGCGGAGATG
oAfHapX-C286A.r	CATCTCCGCGCAAACGGCCGGCGTGCCATCCGAAC
oAfHapX-C350A.f	CAACGGGCCGGGCACAGCTGCGCAGTGTCTTGCAG
oAfHapX-C350A.r	CTGCAAGACACTGCGCAGCTGTGCCCGGCCCGTTG
oAfHapX-C353A.f	CCGGGCACATGCGCGCAAGCGCTTGCAGATCCGCGGAGG
oAfHapX-C353A.r	CCTCCGCGGATCTGCAAGCGCTTGCGCGCATGTGCCCGG
oAfHapX-C380A.f	CTGCCCCGTCGGGATGCGCAGGAGGTAAAGGCGCGG
oAfHapX-C380A.r	CCGCGCCTTTACCTCCTGCGCATCCCGACGGGGCAG
oAfHapX-C389A.f	AAGGCGCGGATGGCGGGGCATGCCAGTCGCGTAACAC
oAfHapX-C389A.r	GTGTTACGCGACTGGCATGCCCCGCCATCCGCGCCTT
oAfHapX-C422A.f	CATCCTTGACTTTATCTGCAGCCGACGCCTACACGAC
oAfHapX-C422A.r	GTCGTGTAGGCGTCGGCTGCAGATAAAGTCAAGGATG

(D) Generation of truncated *hapX*. (^{aa}, amino acids in length of truncated HapX protein)

hapXtrunc-f	GAATGCACAGGTACACTTG	HapX^{aa}
hapX464r	TTAATTAATTCGAATCACCCACGATCGGTTAAGGG	HapX^{464}
hapX398r	TTAATTAATTCGAATCACGAAACATTGGTGTTACGC	HapX^{398}
hapX374r	TTAATTAATTCGAATCAAGCGCTAGGGGAGCGGGA	HapX^{374}
hapX296r	TTAATTAATTCGAATCATTCCTCCTGAGCGGCCAT	HapX^{296}
hapX158r	TTAATTAATTCGAATCATCTTACAGCCTCCCTGTC	HapX^{158}

(E) Generation of Venus tagged HapX. (overlapping complementary sequences are in same color)

5'hapX-f	TGCCTAGTGAATGCTCCG
5'hapXvenus-r	CTCGCCCTTGCTGACCATGGTTACGGATGATGAGACT
5'hapXvenus-f	AGTCTCATCATCCGTAACCATGGTCAGCAAGGGCGAG
venus-r	GTTAATGCAGCTGGCACG
venushapX-f	TGTACAAGGGCGCCAGATCTATGTCTACACCTTCAATAGC
hapX3'-r	ATATCACGAGTCCGTTTGG
5'hapX-f2	ACAACTCTCCTATGAGTCG
venushapX-r	GTAGACATAGATCTGGCGCCCTTGTACAGCTCGTCCATG
hapXtrunc-f	GAATGCACAGGTACACTTG
hapX-r	TTACTTAATTCGAATCATTTGTCGGCAAACCGTCG

(F) Generation of DIG-labeled hybridization probes.

oAfccc1-1.f	GATTCCGACACCCTAGAC	*cccA*
oAfccc1-1.r	GCGATGATGTTGTCCCTG	
hemA-FG1	ATGACTAGTGGTGCACCTG	*hemA*
hemA-FG2r	CTAGGGTGATCTAGATGTTC	
oAfLeu1-f	GGTCTCACATACTTCGGC	*leuA*
oAfLeu1-r	TTCACGCACATCGGCAAG	
oAfhapX2-seq	TAATGTCCATCCAGATCGC	*hapX*
oHapX-seq2	CGAGTCCGTTTGGGTATC	
oAfmirB1me	AAGCCGAGAAAAAGGGGG	*mirB*
oAfmirB2me	AACCCAGATGAAGCCCAG	
oAfAT1me	ACAATCAAGGCTCAGCCC	*sidG*
oAfAT2me	ACTTCGAGTCATGCTGGG	
oAfcytC1	CCCTTTCTTGCAGTGTCC	*cycA*
oAfcytC2	CCGCGCATCTGCTTTTAC	

(G) Surface plasmon resonance analysis (CCAAT boxes are underlined).

BcccAp-369-37	Biotin-AAATCCACCAATCAACGCCTCCGTGATTGTCAGCAAT
cccAp-369-37	ATTGCTGACAATCACGGAGGCGTTGATTGGTGGATTT
BcccAp-369-34	Biotin-AAATCCACCAATCAACGCCTCCGTGATTGTCAGC
cccAp-369-34	GCTGACAATCACGGAGGCGTTGATTGGTGGATTT
BcccAp-369-32	Biotin-AAATCCACCAATCAACGCCTCCGTGATTGTCA
cccAp-369-32	TGACAATCACGGAGGCGTTGATTGGTGGATTT
BcccAp-369-28	Biotin-AAATCCACCAATCAACGCCTCCGTGATT
cccAp-369-28	AATCACGGAGGCGTTGATTGGTGGATTT

(H) ChIP analysis of *cccA* and *actA*.

cccA-ChIPf	CCTCTAGACACTTATAAGGATTTCACATTGTGT
cccA-ChIPrev	CGGATTATCATAACACAGACCACCAG
actA-ChIPf	TGTTGTGTAGGAGGGAGAGAAGT
actA-ChIPrev	TTTCTTTTCCTTGGACCAGTCGC

(I) qRT-PCR analysis of *hapX, sreA* and *actA*.

actA-qPCRf	CCACGTCACCACTTTCAACTC	*actA*
actA-qPCRr	CTGCATACGGTCGGAGATAC	
hapX-qPCRf	GCCTACACGACCCTCTCACG	*hapX*
hapX-qPCRr	GCCCATCACACTCGCAGCC	
sreA-qPCRf	GGCAGTGCGAAGAAGCGAAC	*sreA*
sreA-qPCRr	GGGGGAAGGATTGGCAGTTG	

```
                                                     CBC binding domain
AnHapX    MA--AQPALAIAPSAAPLAPALVAKPTVSPSPGPGTPGSVTSKEWIIPPRPKPGRKPATD 58
AfuHapX   ---MSTPSIAPAPAP--LVPALAAKPAISPSPGPGTPGSITSKEWVIPPRPKPGRKPATD 55
AoryHapX  -MATPAPSIAPAPTPAPLAPAIAAKPTISPSPGPGTPGSVTSKEWVIPPRPKPGRKPATD 59
NfiHapX   ---MSTPSIAPTPAP--LVPALAAKPAISPSPGPGTPGSITSKEWVIPPRPKPGRKPATD 55
AflaHapX  -MATPAPSIAPAPTPAPLAPAIAAKPTISPSPGPGTPGSVTSKEWVIPPRPKPGRKPATD 59
AnigHapX  MATPAAPSLAPAPGPAPLAPALAAKPAISPSPGPGTPGSVTSKEWVIPPRPKPGRKPATD 60
AclaHapX  ---MSAPSMAPTPAPASLAPALAAKPAVSPSPGPGTPGSITTKEWVIPPRPKPGRKPATD 57
             . *::* :*  .  *.**:.***::***********:*:***:***************

                  b(ZIP)                         Coiled coil      115
AnHapX    TPPTKRKAQNRAAQRAFRERRAARVSELEDQIKCIEDDHEIHVATFKEQIANLSREVEQC 118
AfuHapX   TPPTKRKAQNRAAQRAFRERRAARVNELEEQIKKIEDEHEIHIAAFKEQITNLSREVEQC 115
AoryHapX  TPPTKRKAQNRAAQRAFRERRAARVNELEDQIKKIEEEHDIHVAAFKEQISNLSHEVEQC 119
NfiHapX   TPPTKRKAQNRAAQRAFRERRAARVNELEEQIKKIEDEHEIHIAAFKEQITNLSREVEQC 115
AflaHapX  TPPTKRKAQNRAAQRAFRERRAARVNELEDQIKKIEEEHDIHVAAFKEQISNLSHEVEQC 119
AnigHapX  TPPTKRKAQNRAAQRAFRERRAARVNELEDQIKKIEDDHEIHVLQLKEQISNLSHEVEQC 120
AclaHapX  TPPTKRKAQNRAAQRAFRERRAARVNELEDQIKKIEDEHDIHIAAFKEQIGNLSCEVEQC 117
          *************************.***:*** **::*:**:  :**** *** *****

                     126                          158
AnHapX    RTEMGWWRDRCHALEKEVSVERAARETLVKELRSSLPEKNTSGTDAVPLP-----PRSSR 173
AfuHapX   RSEMTWWRDRCHALEKEVSVERSAKEAIVKEFRSSLSDREAVRSDKGLAPLTTSTPQARS 175
AoryHapX  RNEMTWWRDRCHALEKEVSVERSAKEALVKEFRSSLSDKNAP---AGRAPLTRVSARNSG 176
NfiHapX   RSEMTWWRDRCHALEKEVSVERSAKEAIVKEFRSSLSDRNAASSDKGLLPLATST-QNRS 174
AflaHapX  RNEMTWWRDRCHALEKEVSVERSAKEALVKEFRSSLSDKNAP---AGRAPLTRVSARNSG 176
AnigHapX  RSEMAWWRDRCHALEKEVSVERSAKESLVKEFRSSLSDKNASRSDKAPLTR---IPRDSS 177
AclaHapX  RSEMTWWRDRCHTLEKEVSVERSAKEALVKEFRLSLSDKNAASSDAVPFPRATSS----- 172
          *.** *******:*********:*:*::***:* **.::::        .

                                             CRR-A
AnHapX    SSRMELEKSSPVDRRSELG-------EEVPLGCNRCSTSHCQCIEDAF-GMPPIEMNRAP 225
AfuHapX   SDRPDN--GDASNNDSGEG------REEVPLGCNDCSTSHCQCIEDAF-TMPGVVAQEQS 226
AoryHapX  SGRATNERSSPSNANSGSN---DDEQEEVPLGCPSCSSTHCQCIEDAF-AMPGVES-LHS 231
NfiHapX   SERPDN--GDASNNDSGEG------REEVPLGCNDCSTSHCQCIEDAF-TMPGVVAQEQS 225
AflaHapX  SGRATNERSSPSNANSGSN---DDEQEEVPLGCPSCSSTHCQCIEDAF-AMPGVES-LHS 231
AnigHapX  AAHAAVENN-----MHEER-------EEVPLGCSNCSNVHCQCIEDAFSGMPGIERTSAQ 225
AclaHapX  --RPEND-GVPANNASHDDGHHSDNREEVPLGCNDCSTSHCQCIEDAF-AMPGVLAQEES 228
             :     .                *******  **. *********   ** :

                                                                CRR-B
AnHapX    ------------EPKIKPEPEEMEIDFTTRFAAPHHEEDTAASPVASPPVDPCGFCQDGT 273
AfuHapX   RR-LDTTKPGLSEPQIKPDPEEMEIDFTSRFAATQ-QQDQSPTSVSSPAVDPCGFCSDGT 284
AoryHapX  KR-LSTTGQGRAEPEIKPDPEEMEIDFTTRFAAPQ-PQEDNATAVSSPAVDPCGFCQDGT 289
NfiHapX   RS-LDTTKPGRSEPQIKPDPEEMEIDFTSRFAAIQ-QQDHSPTSVSSPAVDPCGFCSDGT 283
AflaHapX  KR-LSTTGQGRAEPEIKPDPEEMEIDFTTRFAAPQ-PQEDNATAVSSPAVDPCGFCQDGT 289
AnigHapX  SKRPDNSSQSHAEPEIKPEPEEMEIDFTTRFAAPHHVQDDTVTNVSSPAVDPCGFCQDGT 285
AclaHapX  RR-PGSTKPGRLEPEIKPDPEEMEIDFTARFATMK-PQDHSPTSVSSPAVDPCGFCQDGT 286
                      **:***:*********:***:  :   ::    : *:**.*******.***

                     296
AnHapX    PCICAEMAAQEEERRRNSTFESNRLAPIQNISQFTPPPSDSDVRSDNVTLPPISQATAAN 333
AfuHapX   PCICAEMAAQEEQRPRRNSFENNRLAPIQNLSQFTPPPSDGDVRS-DVTLPPISQAT--N 341
AoryHapX  PCICAEMAAQEEQ--RRQSFENNRLAPIQNLSQFTPPPSDGDVRS-EVTLPSINQAT--N 344
NfiHapX   PCICAEMAAQEEQRPRRNSFENNRLAPIQNLSQFTPPPSDGDVRS-EVTLPPISQAT--N 340
AflaHapX  PCICAEMAAQEEQ--RRQSFENNRLAPIQNLSQFTPPPSDGDVRS-EVTLPSINQAT--N 344
AnigHapX  PCICAEMAAQEEERRRRNSFENNRLAPIQNLSQFTPPPSDGDVRS-EVTLPPISQAT--N 342
AclaHapX  PCICAEMAAQQEENERRNTFENNRLAPIQNMSQFTPPPSDGDVRS-EMTLPSLSQAT--N 343
          **********:*:    *..:**.********:*********.****  ::***.:.***   *

                  CRR-C             374      CRR-D           398
AnHapX    PCANGPGTCAACLSDPRRTLFCKTLAASRSASGTPSGCCGGKGRDGGCCQSQSRTSAPRR 393
AfuHapX   PCANGPGTCAQCLADPRRTLFCKTLAASRSPSAAPSGCCGGKGADGGCCQS--RNTNVSR 399
AoryHapX  PCANGPGTCAQCLADPKSTLFCKTLAASRSASVASSGCCGGKGADGGCCQS--RSSNPPR 402
NfiHapX   PCANGPGTCAQCLADPRRTLFCKTLAASRSPSAAPSGCCGGKGADGGCCQS--RNTNVSR 398
AflaHapX  PCANGPGTCAQCLADPKSTLFCKTLAASRSASVASSGCCGGKGADGGCCQS--RSSNPPR 402
AnigHapX  PCANGPGTCAQCLADPRRTLFCKTLAASRPASAAPSGCCGGKGADGGCCMS--RNSNPQR 400
AclaHapX  PCANGPGTCAQCLADPRSTLFCKTLAASRSASGTPSGCCGGKGAGGGCCQS--RSSNPSR 401
          ********** **:**: ***********..* :.******** .**** *  *.:   *
```

```
                                    422
AnHapX    --------SNTDRSATP-LTLSCADAFTTLSRHPNFSRASDELASWLPKLHTLPNPRDVS 444
AfuHapX   GRSGSNNNTSSGSSAAPSLTLSCADAYTTLSRHPNFSRATDELSTWLPKLHTLPKPRDFP 459
AoryHapX  ---AAAAKSTSGRSTTPSLTLSCADAFTTLSRHPNFSRASDEISTWLPKLHTLPNPKDVA 459
NfiHapX   GRSGSNNNSTSGSSAAPSLTLSCADAYTTLSRHPKFSRATDELSAWLPKLHTLPKPRDFP 458
AflaHapX  ---AAAAKSTSGRSTTPSLTLSCADAFTTLSRHPNFSRASDEISTWLPKLHTLPNPKDVA 459
AnigHapX  --------SGSRRSATPSLTLSCADAFTTLSRHPNFSRASDDISSWLPKLHTLPNPS-QS 451
AclaHapX  SGSTGNANSIPGPSTTPSLTLSCADAFTTLSRHPSFTRATDDISNWLPKLHTLPNPRDIP 461
                  : .  *::* ********:*******.*:**:*::: *********:*   .

               464
AnHapX    QT---TPASRAAMEVEAASVMGVLRYFDRRFADK 475
AfuHapX   LTD--RGVPRAALEVEAASVMGVLRYFDRRFADK 491
AoryHapX  SPD--RCSSRAALEVEAASVMGVLRYFDRRFADK 491
NfiHapX   LND--RGVPRAAMEVEAASVMGVLRYFDRRFADK 490
AflaHapX  SPD--RCSSRAALEVEAASVMGVLRYFDRRFADK 491
AnigHapX  QKEVMQPGSRAALEVEAASVMGVLRYFDRRFADK 485
AclaHapX  LSD--RGTPRAAMEVEAASVMGVLRYFDRRFADK 493
                  .***:*********************
```

Figure S1. Alignment of *Aspergilli* HapX orthologs. The three single Cys 115, 126 and 422 as well as CRR-A, -B, -C, -D are conserved in the seven HapX orthologs. An, *A. nidulans*; Afu, *A. fumigatus*; Aory, *Aspergillus oryzae*; Nfi, *Neosartorya fischeri*; Afla, *Aspergillus flavus*; Anig, *Aspergillus niger*; Acla, *Aspergillus clavatus*.

```
                                                         CBC binding
AfuHapX MSTP---SIAPAPAPLVPALAAK------------------------------------- 20
AnHapX  MAAQPALAIAPSAAPLAPALVAK------------------------------------- 23
CaHap43 -MPAKGPNIIPKQAPSPIAIAGS------------------------------------- 22
UmYap1  MSSPAMAQILPNPATSSSSIAAQRNLAQRPLVSSSAAASSLKPAPSKPPSSRLAPAPSSS 60
          .     * *  *.   ::...

                                                         CBC binding
AfuHapX ----PAISPSPGPGTPGS--------------------------------ITSKEWVIPP 44
AnHapX  ----PTVSPSPGPGTPGS--------------------------------VTSKEWIIPP 47
CaHap43 ----PPSSSASTPRSAASPPVSVNTKYSPNTNSNTIMPRQVMSI------QTSKEWVLPP 72
UmYap1  IAAAPLRSAAPSPPKPKPATLAPTLSAAPVPSTSTASPVAKKDTSGAPLVQPSKEWVLPA 120
            *  *.:. *  .. .                                 .****::*.

        domain               b(ZIP)                  Coiled coil
AfuHapX RPKPGRKPATDTPPTKRKAQNRAAQRAFRERRAARVNELEEQIKKIEDEHEIHIAAFKEQ 104
AnHapX  RPKPGRKPATDTPPTKRKAQNRAAQRAFRERRAARVSELEDQIKCIEDDHEIHVATFKEQ 107
CaHap43 RPKPGRKPSVDTPASKRKAQNRAAQRAFRERRATRVQELEQKLMEVEKERDIKEMALVNT 132
UmYap1  RAKPGRKPSETEPLTKRKAQNRASQRAFRERKQSYLAELEAKVAAYEAAEIDRSVEIQKV 180
        *.******:   * :********:*******: : : *** ::   *  .  :   : :

                 115        126
AfuHapX ITNLSREVEQCRSEMTWWRDRCHALEKEVSVER---------SAKEAIVKEFRSSLSDRE 155
AnHapX  IANLSREVEQCRTEMGWWRDRCHALEKEVSVER---------AARETLVKELRSSLPEKN 158
CaHap43 INKLKVENQFLVKNLEQLKGEMNQMKQSFSRSQQQLISKSSPAASTASAQQIKPTFSFAS 192
UmYap1  AQKLRAENDSLRKEVSAWKEKFAQIEKYLLQAKANGGRLPGITPSNRSGPGCKAPAHERS 240
          :*  * :   .::   : .   ::: .   :         :.        :..    .

AfuHapX AVRSDKGLAPLTTSTPQARSSDR-----------------------------------PDNG 182
AnHapX  TSGTD--AVPLPPRS--SRSSRM-----------------------------------ELEK 181
CaHap43 NSAPSSHPSPSHHNSISPTGSSYSVQQISPAPSTDSPPNSYNLSNDSYRQFHSTLTPISN 252
UmYap1  SAVPKRGVLIAPGAKSERDRERSAGGVRFADAKPDKP------------------ALQAG 282
           ..         .     .

                    CRR-A
AfuHapX DASNNDSGEGREEVPLGCNDCSTSHCQCIEDAFTMPGVVAQEQSRRLDTTKPGLSEPQ-- 240
AnHapX  SSPVDRRSELGEEVPLGCNRCSTSHCQCIEDAFGMP---PIEMNR--------APEPK-- 228
CaHap43 NNTPDKPLSAADVANFDCGVCPKEECLCESVGLKEPTKDKTENAKKKLQDQVNSFKPMPA 312
UmYap1  SSTEEQQAASVPLRPKPIGVSLPVPPPDAVDTIPSASLDDTASDDQGSLPETGKVAPRKA 342
        . . :             . .           :  .                    *

AfuHapX -----------IKPDPEEMEIDFTSRFAATQQ----------------QDQSPTS-VSSP 272
AnHapX  -----------IKPEPEEMEIDFTTRFAAPHH----------------EEDTAASPVASP 261
CaHap43 VSL---SRKRKIKSTDEEQEIDFTKQFSVKAKPMPDLKKLKKTTFSQPQTETASTFNEDS 369
UmYap1  SKLPLWSFESTKPPSPDAPSLTISATTSSDDKP-----------TSLLSPSLMPQGGHLS 391
                     .  :  .: ::   :   :                . .  .     .

                CRR-B
AfuHapX AVDPCGFCSDGTPCICAEMAAQEEQR----------------------PRRNSFENNRLA 310
AnHapX  PVDPCGFCQDGTPCICAEMAAQEEER----------------------RRNSTFESNRLA 299
CaHap43 PVDNCGFCSDDTPCVCREAAKEAAKLNEYLNNPHQSSIM---------EEEISEEAKTLP 420
UmYap1  VGGGCGFCTEASPCVCADDFLDLSTQTPPSHIAVISDVASAVPLPKRRPASGASETSRRM 451
          . **** : :**:* :   :              NES             : * .
              399     407
AfuHapX PIQN----------------------LSQFT----PPPSDGDV-------------RSD- 330
AnHapX  PIQN----------------------ISQFT----PPPSDSDV-------------RSDN 320
CaHap43 PLQTNNPNHNFSKSALPVMHPGPTVEIREFTNINAVPNVLPAS-------------KNEN 467
UmYap1  SIGSLTHANDLALDQRRRTASNGTGKKLWYTVTQPASPPGYSVTAAVPLSLKSTSKGNPS 511
        .: .                         :*     .                    .

                      CRR-C
AfuHapX VTLPPISQAT--NPCANGPGTCAQCLADPRRTLFCKTLAASRSP------------SAAP 376
AnHapX  VTLPPISQATAANPCANGPGTCAACLSDPRRTLFCKTLAASRSA------------SGTP 368
CaHap43 EKEESTKDTSSDGGCTGNPGTCRQCQMDPMSTLFCTTVASRSTK------------SDSV 515
UmYap1  KKLWTVTETPAEPVCTGDPSTCGACSTDPGLAAFCEAVTTATASPSSSVPSSAAATASSL 571
         .  . .::.    *:..*.**  *  **  : ** ::::  :             :  :
```

```
              CRR-D
AfuHapX SGCCGGKGADGGCCQSR---------------------------------NTNVSRGRSG 403
AnHapX  SGCCGGKGRDGGCCQSQ---------------------------------SRTSAPRRSN 395
CaHap43 SSTRQSISRTNSKTSIS---------------------------------IDSLNNPQSP 542
UmYap1  SPSRPGMSRSGTTGQLLPPYPSRGETIPSAWRQIRSHPRFSQWQGGLDLLAEVVSKRSGN 631
        *    . .  .   .                                           .

                            422
AfuHapX SNNNTSSGS---SAAPSLTLSCADAYTTLSRHPNFSRATDELSTWLPKLHTLP-----KP 455
AnHapX  TDR---------SATP-LTLSCADAFTTLSRHPNFSRASDELASWLPKLHTLP-----NP 440
CaHap43 IPPPLLANN---KTGGSNAPTPTPAPSTPSHSSSVSSNSGIFIPCADAYKTLSRHKKFNS 599
UmYap1  VHSPLLSSKRPREASVEIEPSRSNTISATSNDRATAKPLLLHTTSDTTVHTVP-QIKTED 690
                    .:      : : : :: *.    :       .     :*:.     :

AfuHapX RDFPLTDRGVPRAALEVEAASVMGVLRYFDRRFADK------------------------ 491
AnHapX  RDVSQTTP-ASRAAMEVEAASVMGVLRYFDRRFADK------------------------ 475
CaHap43 VDFTTLVGKLTTRGMQVEVQSVANVLRELDRRAYN------------------------- 634
UmYap1  EDDGLDAREQKRRRILIDREAVQEALALLDAGTAAQPRAGASLSTNPGTGKEEQPCPCPW 750
         *            : ::  :*  .*  :*

AfuHapX --------
AnHapX  --------
CaHap43 --------
UmYap1  RPGERRTP 758
```

Figure S2. Alignment of *A. fumigatus*, *A. nidulans, C. albicans* HapX orthologs and *U. maydis* Yap1.

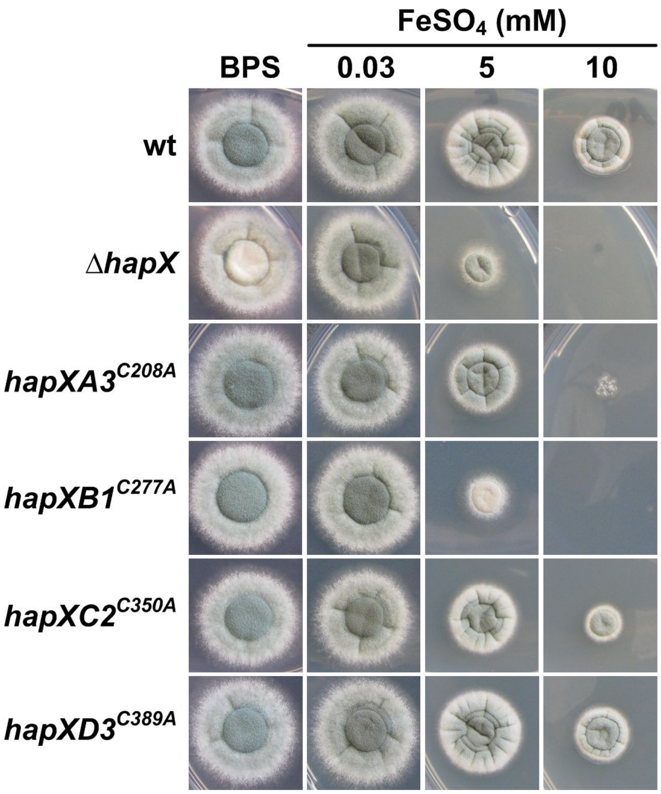

Figure S3. Cysteine-to-alanine mutations within the same CRR cause an identical phenotype (compare to Fig. 4). Strains were grown for 48h at 37°C on agar plates with the given iron concentration. Strains are derivatives of *A. fumigatus* AfS77.

Species	Strand	Start	*p*-value	Sites
Trichophyton equinum	-	489	2.76e-14	GCAGCCAATAGGCGGCCCTCTTATTGTAAGC
Trichophyton tonsurans	-	491	2.76e-14	GCAGCCAATAGGCGGCCCTCTTATTGTAAGC
Trichophyton rubrum	-	481	2.76e-14	GCAGCCAATAGGGGGCCCGCTTATTGTAAGC
Trichophyton verrucosum	-	580	6.32e-14	GCAGCCAATGGGGGGCCCGCTTATTGTAAGC
Arthroderma benhamiae	-	582	7.40e-14	GCAGCCAATGGGCTGCCCGCTTATTGTAAGC
Paracoccidioides brasiliensis	-	528	6.28e-13	GCAGCCAATCAGCACCGCTGGGAATGTCAGC
Arthroderma otae	-	455	7.14e-13	GCAGCCAATAGGAGGGCAGATGATTGTAAGC
Aspergillus flavus	-	370	1.04e-12	CCGGCCAATCACATCTCCGTTAATAGTCAGC
Aspergillus oryzae	-	369	1.04e-12	CCGGCCAATCACATCTCCGTTAATAGTCAGC
Paracoccidioides lutzii	-	523	3.77e-12	GCAGCCAATCAGCACCGTTGGGAATGTAAGC
Ajellomyces dermatitidis	-	369	3.77e-12	CCAGCCAATCAGCACAGCGATAAATGTAAGT
Aspergillus kawachii	-	317	3.77e-12	CGGGCCAATCACAAGCCGCGTAATTGTCAGC
Arthroderma gypseum	-	486	1.08e-11	CCAGCCAATAGAGGGTGCGCTGATTGTAATC
Aspergillus niger	-	315	1.08e-11	TGGGCCAATCACAAGCCGCGTAATTGTCAGC
Penicillium chrysogenum	+	535	1.45e-11	GCAGCCAATCACATCACAGGCAAAGGTCAGC
Aspergillus terreus	-	307	2.12e-11	CGAGCCAATCAGCATTGCGGTAATGGTCAGC
Talaromyces marneffei	-	441	3.07e-11	TCAGCCAATGGGAGAAACTGTAATTGTAATC
Uncinocarpus reesii	-	434	4.37e-11	GCACCCAATCAAAGCAATGCTGATTGTAAGC
Ajellomyces capsulatus	-	346	7.28e-11	CCGGCCAATCAGCACGGCGGTAAATGTAAAG
Neosartorya fischeri	-	532	1.09e-10	TCCACCAATCAACGCATCTGTGATTGTCAGC
Penicillium digitatum	+	609	1.18e-10	GCAGCCAATCGCATCAGCTGCAAAGGTCATC
Aspergillus fumigatus	-	365	1.49e-10	TCCACCAATCAACGCCTCCGTGATTGTCAGC
Coccidioides posadasii	-	415	2.17e-10	CAGGCCAATAGCGTCACAGCAAATAGTAAGC
Coccidioides immitis	-	417	2.17e-10	CAGGCCAATAGCGTCACAGCAAATAGTAAGC
Talaromyces stipitatus	-	467	2.89e-10	TTAGCCAATGAAAACCGTTATAAATGTAAGC
Fusarium oxysporum	-	281	3.11e-10	CCAGCCAATCACGAAGCATGTTTTAGTAATC
Aspergillus clavatus	-	382	3.11e-10	CTGGCCAATCAGCGAGTGCATAATTGTCAGC
Aspergillus nidulans	-	475	6.50e-08	CGAGCCAATCAGAGCGAAGTGATTACCAACC

Figure S4. Identification of an evolutionary conserved motif in the *cccA* promoter regions of 28 fungal species. Sites identified by MEME analysis and their relative positions to the ATG start codon of the CccA coding sequences are shown.

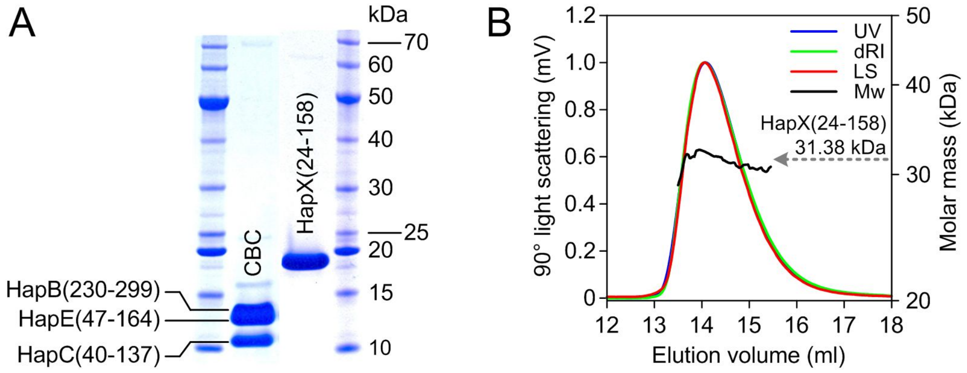

Figure S5. Characterization of purified recombinant proteins used in this study. (A) SDS-PAGE analysis of the CBC and HapX(24-158). (B) Analysis of the solution oligomeric state of HapX(24-158) by size exclusion chromatography and multiangle static light scattering. The light scattering signal (LS) is shown overlaid with the calculated molar mass (Mw) across the elution profile monitored by the absorbance at 280 nm (UV) and changes of the refractive index (dRI).

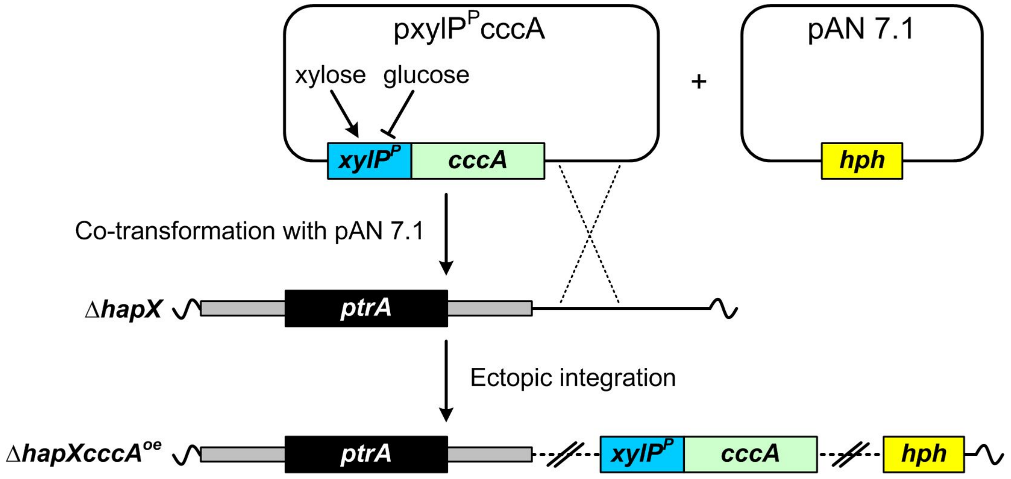

Figure S6. Ectopic integration of inducible *cccA*. pxylP^PcccA was co-transformed with pAN 7.1, a plasmid containing a hygromycin resistance cassette (*hph*) for selection of transformants.

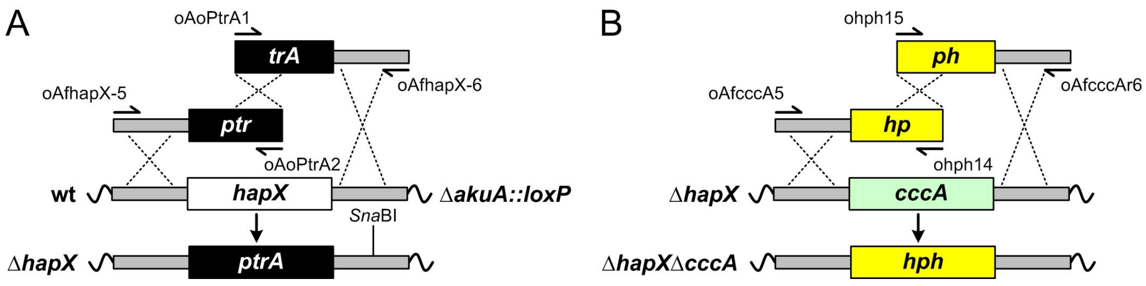

Figure S7. Scheme of *hapX* (A) and *cccA* (B) disruption using the bipartite technique.

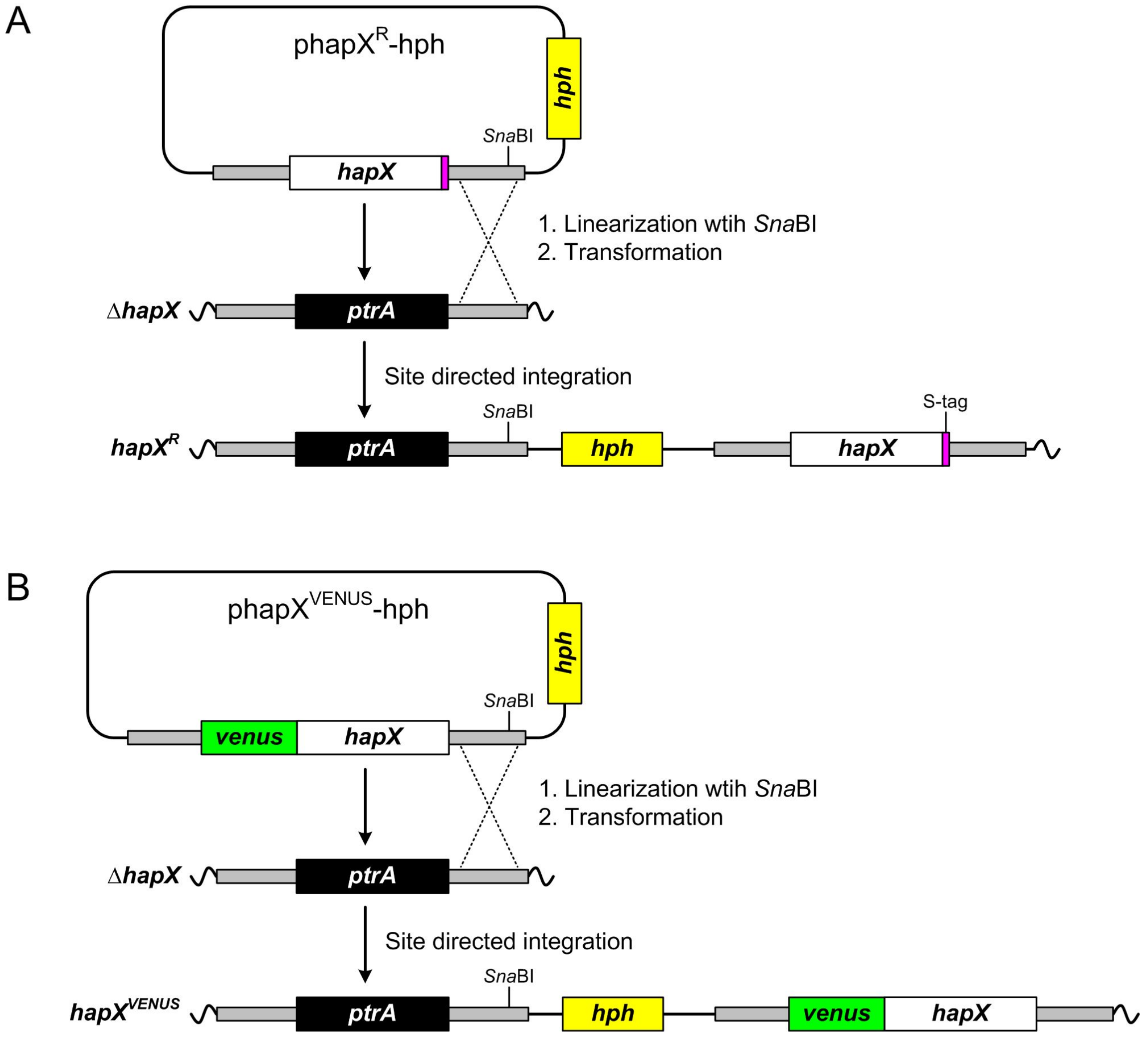

Figure S8. Scheme of site-directed integration of *hapX* plasmids. (A) Site-specific integration is illustrated for plasmid phapX^{R}-hph, which was transformed into Δ*hapX*. The same transformation strategy was performed with plasmids of modified *hapX* (mutations and truncations). (B) Site-directed integration of N-terminally *venus-tagged hapX*.
